# A whole genome sequencing study of moderate to severe asthma identifies a lung function locus associated with asthma risk

**DOI:** 10.1038/s41598-022-09447-8

**Published:** 2022-04-02

**Authors:** Diana Chang, Julie Hunkapiller, Tushar Bhangale, Jens Reeder, Kiran Mukhyala, Jennifer Tom, Amy Cowgill, Jan Vogel, William F. Forrest, Zia Khan, Amy Stockwell, Mark I. McCarthy, Tracy L. Staton, Julie Olsson, Cecile T. J. Holweg, Dorothy S. Cheung, Hubert Chen, Matthew J. Brauer, Robert R. Graham, Timothy Behrens, Mark S. Wilson, Joseph R. Arron, David F. Choy, Brian L. Yaspan

**Affiliations:** 1 Genentech, Inc., 1 DNA Way, South San Francisco, CA 94080 USA; 2 Present Address: Maze Therapeutics, 131 Oyster Point Blvd STE 200, South San Francisco, CA 94080 USA

**Keywords:** Genome-wide association studies, Asthma

## Abstract

Genome-wide association studies (GWAS) have identified many common variant loci associated with asthma susceptibility, but few studies investigate the genetics underlying moderate-to-severe asthma risk. Here, we present a whole-genome sequencing study comparing 3181 moderate-to-severe asthma patients to 3590 non-asthma controls. We demonstrate that asthma risk is genetically correlated with lung function measures and that this component of asthma risk is orthogonal to the eosinophil genetics that also contribute to disease susceptibility. We find that polygenic scores for reduced lung function are associated with younger asthma age of onset. Genome-wide, seven previously reported common asthma variant loci and one previously reported lung function locus, near *THSD4*, reach significance. We replicate association of the lung function locus in a recently published GWAS of moderate-to-severe asthma patients. We additionally replicate the association of a previously reported rare (minor allele frequency < 1%) coding variant in *IL33* and show significant enrichment of rare variant burden in genes from common variant allergic disease loci. Our findings highlight the contribution of lung function genetics to moderate-to-severe asthma risk, and provide initial rare variant support for associations with moderate-to-severe asthma risk at several candidate genes from common variant loci.

## Introduction

Asthma is a heterogeneous complex disease characterized by reversible airway obstruction, airway hyperresponsiveness, and variable inflammation. Genome-wide association studies (GWAS) of asthma have identified more than thirty loci associated with asthma susceptibility^1–5^. Many of these loci point to inflammation mediated by type 2 immunity (e.g. *IL13*) and are enriched in regions with histone marks indicating enhancers in immune cells^2^. Though GWAS have successfully uncovered numerous asthma loci, gaps remain in our understanding of the genetics underlying asthma risk.

First, although many studies have been carried out on asthma risk^1^, only a small fraction specifically focused on severe or uncontrolled asthma patients^6–12^. While these patients only constitute 5–10% of all asthma patients^13,14^, they represent more than 50% of healthcare usage (by asthma patients) and have a large unmet medical need^15^. For these reasons, we focused our study on this asthma subgroup. A recent study (Shrine et al.^8^) carried out a GWAS of over 10,000 patients (including > 5000 cases from the UK Biobank) with moderate-to-severe asthma. While they found that the majority of moderate-to-severe risk loci overlapped with asthma risk loci, the presence of loci associated with only moderate-to-severe asthma suggest some distinct mechanisms underlie mild versus severe asthma. In addition, though it is clinically appreciated that the two key traits underlying asthma and asthma severity—lung function and eosinophilic inflammation—likely represent distinct pathways, how the genetics contributing to these traits overlap and influence asthma is yet to be fully explored.

Second, though the majority of asthma GWAS have focused on common variants, several rare variants contributing to asthma risk have recently been identified. In particular, previous reports in mild to severe asthma have identified rare coding variants in *IL33*^16^ and *GSDMB*^17^, though their contribution to moderate-to-severe asthma risk have not yet been tested. We therefore carried out a whole-genome sequencing study (WGS) on moderate-to-severe asthma patients. We aim to identify common and rare genetic contribution to disease risk, and to compare and contrast the role of eosinophil and lung function genetics in moderate-to-severe asthma.

## Results

### Common genetics of asthma risk in moderate-to-severe patients

In this study, we first analyzed common variants obtained from whole-genome sequences of 3181 moderate-to-severe asthma cases and 3590 non-asthma controls of majority European ancestry (fraction of European ancestry > 0.85 as estimated by Admixture^18^). Asthma cases were derived from ten studies, eight of which were clinical trials (see “Methods”). Healthy control samples with comparable sequencing data were unavailable as sequenced samples were mainly from clinical trials. We thus obtained disease controls from participants in non-asthma clinical trials that were sequenced and processed with the same informatic pipeline to minimize batch effects that may be introduced via differing sequencing technologies and bioinformatics processing pipelines (see “Methods”). These non-asthma disease controls comprised 1140 and 2450 patients from clinical trials of age-related macular degeneration (AMD) or rheumatoid arthritis (RA), respectively. Though similar pathways may contribute to both asthma and RA (e.g. IL6^19^ and Th1 cells^20^), there is minimal genetic correlation between the two traits (r_g_ = 0.12, P = 0.06). AMD and asthma similarly have a low genetic correlation (r_g_ = 0.08, P = 0.07). Additionally RA and AMD are not genetically correlated (r_g_ = 0.035, P > 0.3) and share few genetic loci (n = 2) suggesting limited confounding was introduced by the use of RA and AMD as controls in this study (see Supplementary Note S1). Furthermore, to remove potential association signals originating from our controls, we applied a differential-effects test to remove variants with effect sizes that were significantly different (P < 0.01) when comparing asthma cases to AMD controls versus comparing the same asthma cases to RA controls (see “Methods”). This test successfully filtered known AMD and RA loci (e.g. the associations of the *CFH* and *ARMS2*/*HTRA1* loci with AMD) (Supplemental Fig. S2).

Variants were removed for failing an allelic depth balance test, Hardy–Weinberg equilibrium, and/or having high missingness rates (see “Methods”). After variant filtering, there were 7,165,996 common variants (MAF > 1%). We corrected for genetic sex and the top five principal components and observed minimal inflation in the p-values genome-wide (λ_gc_ = 1.062, λ_1000_ = 1.018) (Supplemental Fig. S1). We used a previously reported, independent study, by Shrine et al. 2019 moderate-to-severe asthma risk stage 1 GWAS^8^ (5135 cases and 25,675 controls) to replicate variants discovered in our study. The use of this independent study to replicate our findings further generalize our findings beyond cases studied in a clinical trial setting.

We first estimated the narrow-sense heritability (h^2^) of moderate-to-severe asthma risk in our study using the LD-score regression framework^21^. We assumed a prevalence of 0.0084 for moderate-severe asthma by using a prevalence of 0.084 for all asthma and assuming moderate-severe asthma patients account for 10% of all asthma patients^13^. Using the prevalence above, we estimated that the h^2^ for our moderate-to-severe asthma risk study is 0.29 (s.e. = 0.045) (estimated h^2^ assuming a range of other prevalences are available in Supplemental Table S1). We further estimated the genetic correlation between moderate-to-severe asthma risk as defined in our study and in the previously published Shrine et al. study^8^ to be 0.54 (s.e. = 0.096). This was lower than the estimated genetic correlation between moderate-to-severe asthma risk as defined in our study and general asthma risk as defined in Demenais et al.^2^ (r_g_ = 0.71, s.e. = 0.03) though the 95% confidence intervals of these estimates overlap.

**Figure 1:**
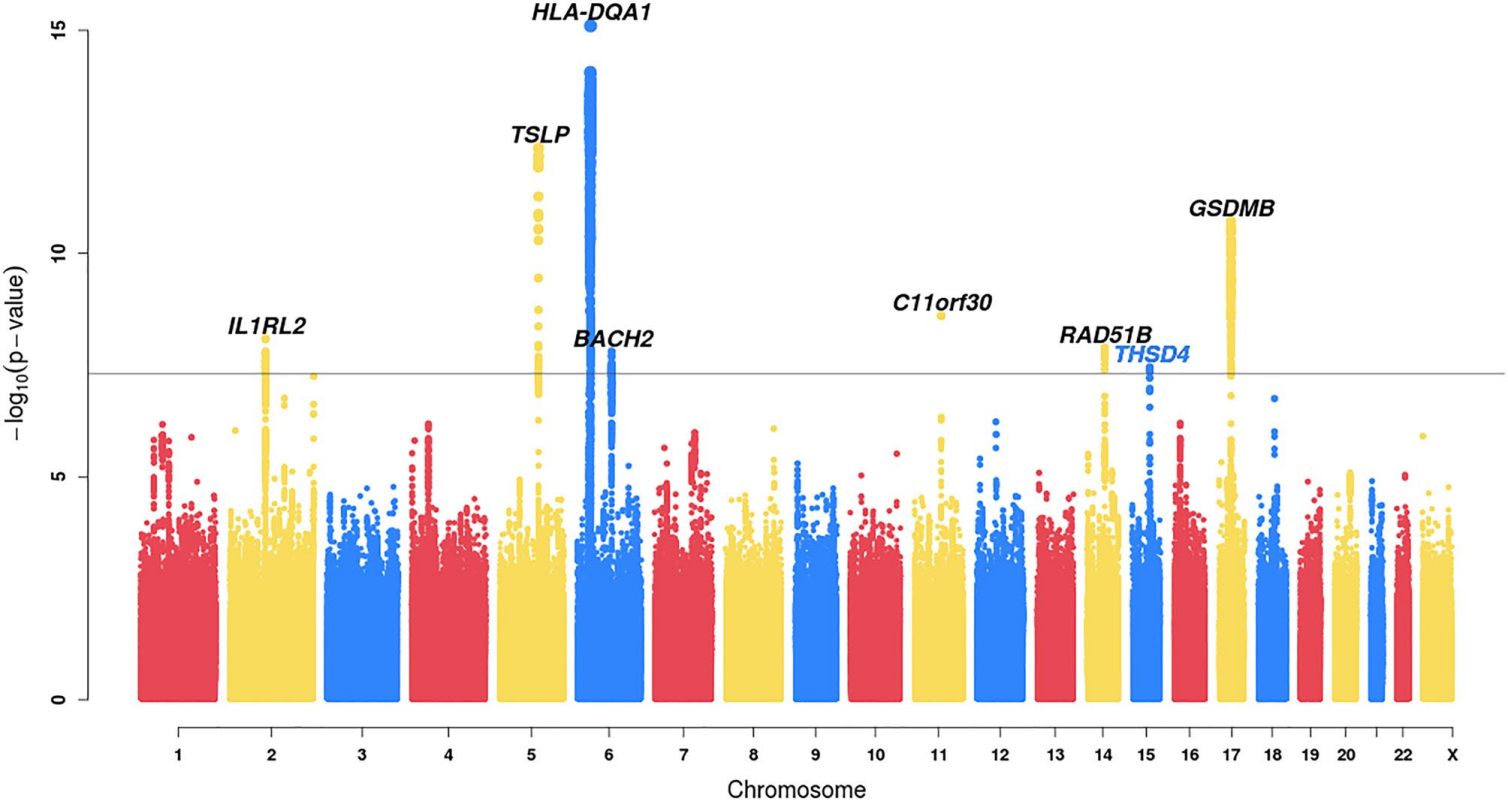
Manhattan plot of common variants (MAF > 1%) associated with asthma risk in this GWAS of 3,181 moderate-to-severe asthma patients and 3590 non-asthma controls. The −log_10_ two-sided p-values are displayed. Variants with P < 0.01 for the differential effects test (See “Methods”) are filtered out. See Supplemental Fig. S2 for a corresponding plot which includes all variants. The black line indicates the genome-wide significance threshold of 5 × 10^–8^. Previously reported loci are labeled in black, the novel locus is labeled in blue.

Eight regions reached genome-wide significance (P < 5 × 10^–8^), seven of which were within 1 Mb of regions previously reported to be associated with asthma (Fig. 1, Table 1). An additional 16 previously reported asthma associations^1,2^ (Supplemental Table S2) and 34 previously reported allergic disease associations^22^ (Supplemental Table S3) showed at least nominal evidence for significance (P < 0.05) in this study. The previously reported moderate-to-severe asthma risk locus near *MUC5AC* showed modest levels of significance with the same direction of effect (rs11603634, P = 2.99 × 10^–3^, OR_G_ = 1.11).

**Table 1:** Sentinel variants in eight regions significantly associated with moderate-to-severe asthma risk in this GWAS of 3181 cases and 3590 controls (OR = odds ratio for coded allele, SE = standard error, P_DE_ = p-value for the differential effects test, RAF = risk allele frequency for AMD and RA controls, respectively).

SNP	CHR	BP (GRCh38)	Nearest gene	Risk/non-risk allele	OR	SE	P-value	P_DE_	RAF_case_	RAF_control_
rs139210940	2	102,265,885	*IL1RL2*	AT/A	1.339	0.051	8.08 × 10^–9^	0.333	0.879	0.847, 0.843
rs10455025	5	111,069,301	*TSLP*	C/A	1.304	0.037	4.36 × 10^–13^	0.856	0.387	0.34, 0.326
rs17205170	6	32,634,706	*HLA-DQA1*	G/T	1.449	0.046	7.92 × 10^–16^	0.168	0.84	0.797, 0.779
rs2875584	6	90,240,909	*BACH2*	C/T	1.238	0.038	1.57 × 10^–8^	0.351	0.706	0.666, 0.653
rs7130588	11	76,559,639	*C11orf30*	G/A	1.242	0.036	2.46 × 10^–9^	0.04	0.386	0.358, 0.332
rs2104047	14	68,287,700	*RAD51B*	T/C	1.245	0.039	1.28 × 10^–8^	0.134	0.312	0.262, 0.274
rs11631778	15	71,314,041	*THSD4*	G/A	1.225	0.037	3.54 × 10^–8^	0.436	0.354	0.305, 0.311
rs7216558	17	39,913,818	*GSDMB*	T/C	1.263	0.035	1.91 × 10^–11^	0.442	0.545	0.479, 0.494

The genome-wide significant association that did not map to any previously reported asthma loci maps to chromosome 15 near the gene *THSD4* (rs11631778, OR_G-allele_ = 1.23, P = 3.54 × 10^–8^, MAF_cases_ = 0.35) (Table 1)*.* The MAF of this variant is higher in our cases as compared to the control samples in our study (MAF = 0.31) as well as in gnomAD (v2.1.1) (MAF_non-Finnish-EUR_ = 0.32) and UK Biobank participants over the age of 50 with no documented respiratory disorders (MAF = 0.33). We were able to replicate association of this *THSD4* variant with moderate-to-severe asthma risk in the Shrine et al. study^8^ (P = 0.0079, OR_G-allele_ = 1.06), but were unable to replicate this association (using the proxy SNP in high LD with our lead variant—rs11853359, r^2^ = 0.93) in studies that did not enrich for moderate-to-severe asthma patients (European subset of the Demenais et al. study^2^, and GWAS of the asthma Phecode in the UK Biobank^23^)(P > 0.45 ).

Conditioning on the index variant in the region (rs11631778) did not reveal any independent associations passing a genome-wide significant threshold of 5 × 10^–8^. Applying FINEMAP^24^ to the region further supported a single causal signal of association with asthma at this locus and found 10 variants in the 95% credible set (Supplemental Table S4), with the lead SNP having a posterior inclusion probability of 0.23. Variants in the 95% credible set overlapped with enhancers and histone marks in lung tissue and lung-related cell-types, and were also associated with expression of *THSD4* in lung samples from GTEx (Supplemental Table S5). To confirm that the lung eQTL and asthma risk association point to the same underlying causal variant, we carried out colocalization analysis via the *coloc* package in R (see “Methods”)^25^. We found there was a high probability of colocalization between the lung eQTL and the asthma risk association (probability_colocalization_ = 0.99) (Supplemental Fig. S4).

Multiple traits can contribute to asthma pathology with lung function (which can be viewed as a proxy for structural changes in the airway leading to variable airflow limitation) and eosinophilic inflammation being two of the major traits^26^. It is now appreciated clinically that these two traits may not be causally linked^27^ and we set out to test whether genetic analyses further support this distinction. The novel moderate-to-severe asthma locus uncovered above is likely contributing to asthma via lung function as the lead SNP, rs11631778, is in high linkage disequilibrium (LD) (r^2^ = 0.95) with a SNP (rs1441358) associated with increased COPD risk^28^, and reduced lung function^29^, but is not associated with eosinophil blood count (P > 0.05)^30^. We next sought to investigate whether this distinction between the two traits extended beyond the *THSD4* locus.

We estimated the genetic correlation between lung function measures and blood eosinophil counts, and asthma risk. We confirmed previous reports^31^ that blood eosinophil cell count and asthma risk are genetically correlated (r_g_ = 0.30, Supplemental Table S6) by applying LD-score regression^21^ to a GWAS of blood eosinophil cell counts in the INTERVAL study^30^ and the Demenais et al. asthma risk GWAS^2^. We found this genetic correlation was also present between moderate-to-severe asthma risk and blood eosinophil cell count (r_g_ = 0.28). We found a similar genetic correlation when using moderate-to-severe asthma risk as defined in Shrine et al.^8^ (Supplemental Table S6). Next, we used a recent meta-analysis of lung function traits carried out on the UK Biobank and SpiroMeta cohorts^29^ of four lung function measures: FEV_1_ (forced expiratory volume in 1 s), FVC (forced vital capacity), PEF (peak expiratory flow) and FEV_1_/FVC^21^ to calculate the genetic correlation between these traits and asthma risk. We found inverse genetic correlation between overall asthma risk and lung function (e.g. higher asthma risk was genetically correlated with lower lung function) (r_g_ ≤ − 0.21 for all lung function traits, Supplemental Table S6). As with overall asthma risk, we found that moderate-to-severe asthma risk was also inversely correlated with all lung function measures (r_g_ < − 0.16) (Supplemental Table S6). This inverse genetic correlation was replicated in the moderate-to-severe asthma risk published by Shrine et al.^8^ (Supplemental Table S6).

We next explored whether lung function genetics overlap with the genetics of blood eosinophil counts or whether they represent independent pathways that may contribute to asthma pathology. While asthma risk shows evidence of shared genetics with both lung function measures and eosinophils, we found that the sharing between eosinophils and lung function measures was low (− 0.086 < r_g_ < − 0.039), suggesting they represent orthogonal axes contributing to overall asthma risk pathology (Supplemental Table S6). Given the distinct variants contributing to these two traits, we next investigated whether these variants influenced the trajectory of asthma—specifically we asked if and how these two axes impacted age of onset in asthma patients. While several risk factors (e.g. allergic sensitization) contribute to childhood onset asthma, proposed risk factors for adult onset asthma include upper respiratory tract infections, exposure to pollutants, hormonal factors, and obesity^32^. Impaired lung function is also observed to be lower in adult onset asthma as compared to childhood onset^33^, though other studies suggest limited impact of lung function on age of onset^34,35^. Therefore, we hypothesized that genetic variants associated with lung function would likely be associated with asthma age of onset.

**Figure 2:**
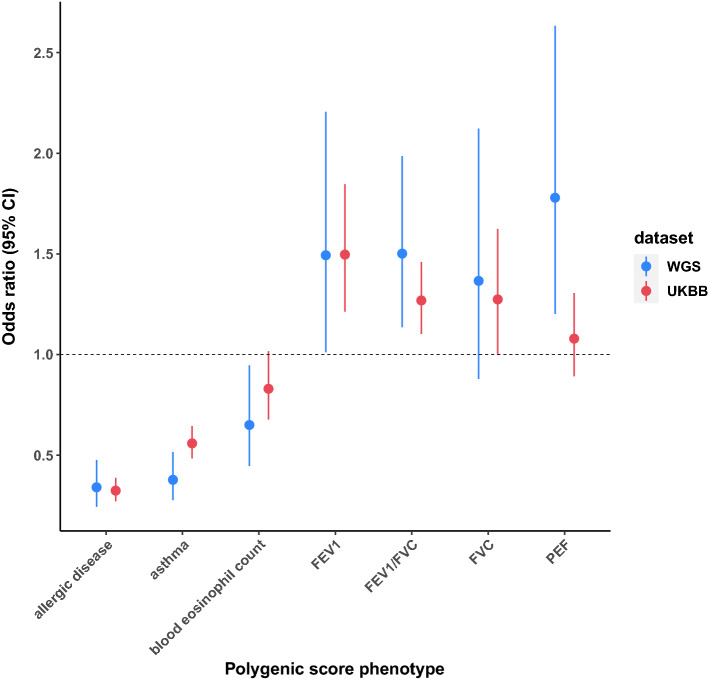
Association of polygenic scores with asthma age of onset. Childhood onset asthma was defined as age onset ≤ 12 and adult onset asthma was defined as age of onset ≥ 25. This binary age of onset trait was regressed on polygenic scores of various traits. Polygenic scores were calculated from publicly available GWAS of allergic disease (n = 242,569), asthma (n = 127,669), blood eosinophil cell counts (n = 40,521) and various lung function measures (n = 400,102). The analysis was carried out in the current whole-genome-sequencing cohort (WGS) (n = 1456) as well as in a cohort of UK Biobank participants (n = 6312). Increased polygenic scores for asthma, allergic disease and blood eosinophil counts are associated with younger age of onset, while increased lung function polygenic scores generally associate with adult onset asthma. Association statistics for PS analyses are available in Supplementary Table S7.

To test this hypothesis, we generated polygenic scores (PS) in a subset of our patients for which age of onset data were available (N = 1456). To briefly summarize polygenic scores, a polygenic score for each individual is calculated by how many phenotype-associated alleles an individual carried weighted by the allele’s effect on that phenotype. A large polygenic score can be interpreted as someone with a higher likelihood, based upon their genetics, to have that phenotype (e.g. asthma) compared to someone with a low polygenic score. We scored individuals using publicly available GWAS summary statistics for allergic disease^22^, asthma risk^2^, blood eosinophil count^30^, and lung function measures^29^ (see “Methods”). We created a binary phenotype, binning patients into whether they had childhood onset asthma (age onset ≤ 12, N = 665) or adult onset asthma (age ≥ 25, N = 791) and regressed this binary phenotype on each PS. As expected, we found that increased PSs for allergic disease and asthma were associated (P ≤ 1.09 × 10^–9^) with childhood onset asthma (OR_allergic_disease_ = 0.34, OR_asthma_ = 0.38) (Fig. 2, ) after correcting for testing seven PS. As hypothesized, PSs for FEV_1_/FVC and PEF were significantly associated with childhood onset asthma (P ≤ 3.97 × 10^–3^), where a higher lung function PS was associated with adult onset asthma or protective for childhood onset asthma. Though the PS for blood eosinophil count was nominally associated with asthma age of onset (P = 0.02), this did not pass multiple testing correction.

To replicate our findings in an independent cohort, we carried out a similar age of onset analyses in 6,312 UK Biobank participants with moderate-to-severe asthma as defined in Shrine et al.^8^ (see “Methods”). We were able to replicate the allergic disease, asthma, and FEV_1_/FVC PS associations with age of onset (P ≤ 9.06 × 10^–4^) (Fig. 2, Supplementary Table S7).

### Rare coding variants associated with moderate-to-severe asthma risk

After variant level QC filters as described above, we were left with 3,600,569 rare (MAF < 1%) exonic variants. Previous studies reported the minor allele of a rare loss of function variant in *IL33* (rs146597587-C) was associated with reduced asthma risk^16^ and the minor allele of a rare missense variant in *GSDMB* (rs12450091-C) was associated with increased risk of asthma^17^. We were able to replicate the association of the *IL33* variant in our study (OR_C-allele_ = 0.37, p-value = 0.025, MAF = 1.93 × 10^–3^), but not the *GSDMB* variant (OR_C-allele_ = 1.32, P = 0.79, MAF = 2.32 × 10^–4^). Genome-wide, no individual rare coding variants were significant after correcting for the number of rare coding variants tested (strict Bonferroni cut-off of correcting for 3,600,569 rare coding variants, P < 1.39 × 10^–8^).

To improve power (especially for genes with extensive allelic heterogeneity), we further aggregated rare variants into gene coding regions. To do so we employed the rare variant burden test as this was compatible with the differential-effects test used in this study to flag results that may be significant due to the use of disease-controls. Burden tests are well powered for scenarios where there is a large fraction of causal variants with a similar direction of effect. On the other hand, we will have reduced power to detect genes with a small fraction of causal variants with opposing directions of effect^36,37^. In the burden test, we coded individuals as 0 or 1 dependent on whether the individual carried a rare allele in that gene. We carried out two sets of burden tests. In the first, we only considered variants with a predicted high impact on protein function (loss-of-function test). In the second, we considered variants in the first test as well variants with a moderate predicted impact that had a PolyPhen^38^ score (probability of the variant being deleterious) > 0.5 (loss-of-function and moderate impact variant test) (see “Methods”). For each burden test, only genes with ≥ 2 variants and ≥ 5 carriers of rare variants satisfying the criteria above were considered for testing. 3166 genes passed our filtering criteria for our first burden test of loss-of-function variants, and 15,836 genes passed our criteria for having loss of function variants and/or moderate impact variants with PolyPhen > 0.5.

Overall, no genes were significant after correcting for the number of genes tested exome-wide (Supplemental Figs. S5, S6). We next sought to test whether collectively they were enriched for association of candidate genes from common-variant allergic diseases, moderate-to-severe asthma and/or lung function GWAS. We investigated three gene-sets corresponding to 132 allergic disease candidate genes, 17 moderate-to-severe asthma candidate genes, and 68 lung function candidate genes for common variant risk loci previously identified^8,22,29^. A majority of these candidate genes reported by the original studies were supported with either coding level or eQTL support linking genes to the associated loci (further details can be found in the “Methods” as well as in the original publications^8,22^). We found significant enrichment for association of genes within the allergic disease gene-set for loss-of-function and moderate impact rare variant burden (P = 0.004) (see “Methods”). We also found nominal significance for enrichment of the lung function gene-set (P = 0.031) though this did not pass multiple testing correction for three gene-sets and two rare variant burden masks (Supplemental Table S8).

Within the allergic diseases and moderate-to-severe asthma gene-sets, *FLG* was nominally significant in the loss-of-function burden test (P = 0.024, OR = 1.473), and eight additional genes passed nominal significance in the high or moderate (PolyPhen > 0.5) impact burden test (*RERE, IQCB1, PPP2R3C, PITPNM2, DYNAP, TSLP, EAF2, RASA2)* (Supplemental Tables S9 and S10). Of the lung function candidate genes, rare variant burden in four genes (*MAPT, CFDP1, EML3,* and *LTBP4*) was nominally associated with asthma risk in our study (Supplemental Tables S9 and S10).

To investigate whether any of these nominally associated genes in our study had rare variant support from an independent cohort we turned to the partially released whole-exome sequencing (WES) data for 200K UK Biobank participants. Of the 6312 participants with moderate-to-severe asthma used in the above common variant analysis, WES data were available for 3418 samples. We compared these to 111,261 control participants with WES data available and no respiratory phenotypes (see “Methods”) and carried out rare variant burden tests as described above for the 13 candidate genes with nominal significance for association with asthma in our study. We used METAL^39^ to carry out a meta-analysis between the UK Biobank data and our study for these genes. Of the 13 candidate genes, only the loss-of-function association for *FLG* became more significant in the meta-analysis (P = 0.0013, OR = 1.30) (Supplemental Tables S9 and S10).

## Discussion

In this study we present a sequencing cohort of patients with moderate to severe asthma. Overall, eight regions reached genome-wide significance in our study, seven of which overlap previously reported asthma risk variants. This is consistent with findings from a previous GWAS of moderate-to-severe asthma that reported significant overlap between variants associated with moderate-to-severe asthma and asthma risk^8^. One possible explanation for this is that the genetic contribution to asthma severity is modest, suggesting a larger role for environmental factors. The remaining genome-wide significant locus in our study mapped to a region containing *THSD4* and only replicated in a study of moderate-to-severe asthma risk^8^. Colocalization analysis supports *THSD4* as the candidate gene for this association signal. *THSD4,* thrombospondin type 1 domain containing 4, is an extracellular matrix protein that is involved in microfibril formation, and may contribute to the structural integrity of the lungs^40^. This locus has previously been associated with lung function and COPD risk^28,29^ and adds to the growing genetic support for the role of lung function determinants in risk of moderate-to-severe asthma.

Clinically, lung function (as a proxy for structural changes to the airway) and eosinophilic inflammation are key components of asthma pathology. Asthma risk loci contributing via the eosinophilic axes have been well appreciated^2,8^ and there is growing support for lung function genetics as well^41^. In this study, we show that while both axes (blood eosinophil cell count and lung function traits) are genetically correlated with asthma risk, the low genetic correlation between eosinophil counts and lung function measures support these as orthogonal axes that contribute to asthma pathology. In other words, the pathways that underlie the eosinophilic axis of asthma biology are likely distinct from those underling lung function determinants. Furthermore, our polygenic scoring analyses highlight a greater contribution of lung function genetics to moderate-to-severe asthma age of onset. Though these pathways may be distinct, it does not rule out that for any one patient both (or neither) pathways may be at play and are contributing to disease risk. Indeed, though both traits show genetic correlation with asthma risk, the correlation is moderate, highlighting the need to uncover additional axes underlying asthma biology.

From the whole-genome sequencing data we were able to assess the rare variant contribution to moderate-to-severe asthma risk. We replicated the association of a previously reported rare variant in *IL33* with asthma risk and found significant enrichment of rare variant burden in candidate genes from common variant allergic disease loci. In a meta-analysis between this study and the partial release of WES data from the UK Biobank of the candidate genes from common allergic disease risk, moderate-to-severe asthma risk, and lung function loci that showed nominal evidence of association in a gene burden analysis of rare variants in our study, only the association with *FLG* became stronger, though the meta-analysis p-value did not meet multiple testing correction. In this study we observed a nominal association between burden of rare variants in *FLG* and increased risk of asthma. This is consistent with the hypothesis that dysfunction in filaggrin’s ability to form and maintain a protective skin barrier can predispose individuals to asthma^42^. Because larger sample sizes are necessary for rare variant discovery^43^, combining sequencing data across moderately sized cohorts is crucial to uncovering rare variants in asthma risk.

There are several limitations to our study. First, we utilized non-asthma disease samples as controls (see “Methods”) and second, our study samples are mostly derived from trial participants which may introduce biases based on various enrollment criteria (see “Methods”). To address the former, we flagged and removed any variants or results that failed to pass the differential effects test throughout this study (see “Methods” and Supplementary Note S1). To address the use of clinical trial samples, we replicated results (when possible) in external independent cohorts that did not have clinical trial-based enrollment criteria. Despite the use of clinical trial asthma participant cases and non-asthma disease controls, we were able to replicate many previously reported common variant asthma risk associations with similar directions of effect.

In summary, we carried out a whole-genome sequencing analysis of moderate-to-severe asthma. We discovered and replicated a common variant association that overlaps a COPD risk and lung function locus. We further provide genetic support for a role of lung function in both moderate to severe asthma risk and age of onset. Finally, our rare variant analyses replicated a previous association in *IL33* and suggest some asthma common variant loci may contain additional rare variant support.

## Methods

### Cohort description

DNA was derived from moderate to severe asthma patients participating in the clinical studies of omalizumab (NCT00252135 [EXCELS], NCT00314575 [EXTRA] and NCT00813748 [X-PAND], lebrikizumab (NCT01545440 [LUTE], NCT01545453 [VERSE], NCT00930163 [MILLY], NCT01867125 [LAVOLTA I], NCT01868061 [LAVOLTA II]), and an additional asthma observational (NCT00091767 [TENOR II]) study and a smaller biomarker study (BOBCAT)^44^. Several of these studies (BOBCAT, EXTRA, LUTE, VERSE, MILLY, LAVOLTA I and LAVOLTA II) had inclusion criteria requiring pre-bronchodilator FEV_1_% predicted to be ≥ 40% and ≤ 80%. Full inclusion and exclusion criteria for all studies (with the exception of BOBCAT) can be viewed at www.clinicaltrials.gov.

Batch effects may be introduced if cases and controls are derived from differing sequencing methods and informatics pipelines. We therefore compared our asthma cases to 1140 and 2450 controls without asthma derived from clinical trial cohorts of AMD^45,46^ and RA^47–58^, respectively that were generated with largely the same sequencing platforms (see Supplementary Note S1 and Supplemental Tables S11 and S13), protocols and also analyzed with the same downstream bioinformatics pipeline.

### Data generation and quality control

Samples were sequenced to an average read depth of 30 × using the Illumina HiSeq platform. Reads were aligned using BWA (version 0.7.9a-r786) to the GRCh38 reference genome (GCA_000001405.15) including alternate assemblies. In regions with alternate assemblies we followed the same alignment and variant calling procedures below, but used an alternate-assembly aware version of BWA (version 0.7.11) to properly handle alignment of reads to reference and alternate-assemblies. After alignment, we followed the GATK best practices guidelines to jointly call variants from WGS data using the Sentieon Genomics pipeline (version 201611.01). While we were able to jointly genotype all samples for exonic variants for rare variant analyses, due to memory and computing time requirements we were unable to jointly call whole-genome data. Therefore, we carried out several batches of joint-genotyping: one batch for asthma cases, and one for each control disease (AMD and RA). We used a merged VCF from these three joint-genotyping runs as input for our whole-genome common variant (MAF ≥ 1%) analyses, without imputing or filling in genotypes missing between batches. We ran a fourth joint-genotyping run on case and control samples together in the exonic regions as input for our rare variant genic analyses.

We filtered variants that did not pass GATK variant quality recalibration threshold of 99% sensitivity and set any genotypes to missing where the genotype quality score was < 20. We further removed SNPs with a missingness rate > 0.05, a Hardy–Weinberg equilibrium p-value < 1 × 10^–6^, and an allelic depth balance test p-value < 0.01. The allelic depth balance test was carried out by testing for equal allele depth at heterozygote carriers via a binomial test. A total of 7,165,996 common variants and 3,600,569 rare exonic variants passed these variant-level QC metrics.

We estimated ancestry using predefined allele frequencies from reference populations in the 1000 Genomes. This approach has been implemented in iAdmix^59^ and the projection function in ADMIXTURE^18^. To minimize confounding due to ancestry, we only retained individuals with fraction of European ancestry > 0.85 for analysis^60^. We further excluded samples with high missingness (missingness > 0.1), relatedness (Z0 ≥ 0.4), excess heterozygosity (≥ 3 standard deviations from the mean), and principal component analysis (PCA) outliers. PCA outliers were defined as at least 6 standard deviations from the mean on any of the top ten principal components, with the outlier removal process iterated five times. Genetic sex was estimated from X-chromosome heterozygosity via PLINK^61^.

### External datasets

Pre-computed summary statistics for lung function measures^29^, moderate-to-severe asthma risk^8^, asthma risk^2^, and allergic disease risk^22^ were downloaded from the GWAS catalog (https://www.ebi.ac.uk/gwas/downloads/summary-statistics). Data for the blood eosinophil count GWAS were downloaded from http://www.bloodcellgenetics.org/. We focused on the INTERVAL study subset of Astle et al.^30^ to avoid any confounding from asthma and COPD patient samples present in the UKBB and BiLEVE cohorts^30^.

### Moderate to severe asthma age of onset analysis in the UK Biobank

This research has been conducted using the UK Biobank Resource under Application Number 44257. We defined moderate-to-severe asthma patients and controls in UK Biobank following Shrine et al. (see Supplemental Methods S1^8^). Briefly, moderate-to-severe asthma cases were defined as having doctor diagnosed asthma and did not report having doctor-diagnosed emphysema or chronic bronchitis (UK Biobank data field 6152). In addition, cases were defined by prescriptions (UK Biobank data field 20003) satisfying the British Thoracic Society and British National Formulary guidelines for moderate to severe asthma. For a full list of medications see Supplemental methods of Shrine et al.^8^. Controls were defined as individuals with (1) no reported diagnosis of asthma, rhinitis, eczema, allergy, or chronic bronchitis/emphysema (UK Biobank data field 6152), (2) were not taking any medication for lung-related conditions, and (3) did not have asthma (J45-J46) nor COPD/bronchiectasis (J40-J44, J47) ICD10 codes.

Samples were removed if they were related or were not of majority European ancestry (fraction European ancestry > 0.70). In the age of onset analysis, 6312 cases were retained after sample quality control and including only those with age of onset data. We had 9,685,491 common variants (MAF > 1%) from which to construct polygenic scores after filtering on Hardy–Weinberg equilibrium (HWE) (P < 1 × 10^–6^), and missingness (missingness > 5%). For the rare variant meta-analysis at candidate genes of common variant loci, we subset to individuals with WES data based upon the October 2020 release of UK Biobank WES data. After sample level QC as described above, we retained 3418 moderate-to-severe asthma cases and 111,261 controls for analysis. We retained 1,759,922 exonic variants with MAF < 0.01, P_HWE_ > 1 × 10^–6^ and missingness > 0.2 as input to our gene-level burden tests.

### Statistical analysis

For single variant association analysis, asthma risk was regressed on genotype assuming an additive genetic effects model. Single variant analyses were carried out in PLINK (version 1.9). For rare variant (MAF < 1%) burden tests, rare variants were aggregated into genic units. Specifically, individuals were scored for their rare allele carrier status and risk status was regressed on this binary rare allele carrier status. The rare variant burden test was carried out in R using the *seqArray* (version 1.18.2) framework to store and access individual genotypes^62^. To enrich for rare variants with impact on protein function, we only considered variants with a “HIGH” (e.g. frameshift, splice acceptor, stop gained, etc., …) and “MODERATE” (e.g. missense variant, inframe deletion or insertion, splice region variant, etc..) predicted impact as annotated with SNPEff (version 4.3q)^63^. We carried out two different sets of burden tests: (1) a stricter set consisting of high confidence variants predicted to have a high impact (as predicted by Loss-of-function transcript effect estimator, LOFTEE version 0.3) (https://github.com/konradjk/loftee) and (2) all SNPEff predicted high impact variants as well as moderate impact variants with a PolyPhen^38^ score > 0.5.

All association analyses were corrected for genetic sex, and the top five PCs. We used FINEMAP (version 1.3.1)^24^ with default settings to estimate 95% credible sets for genome-wide significant associations in our study. Chromatin annotation of variants in the 95% credible set was carried out via *haploR* (version 3.0.1)^64^ to query HaploReg v4.1^65^.

In this WGS-based study, as non-asthma controls samples consisted of AMD and RA patients, we implemented a differential effects test to help filter or flag associations that likely originated from the AMD or RA samples. The intuition motivating the test is that an asthma association will have a similar effect size regardless of the control cohort used in the GWAS (see caveats below). For each variant and association test, we therefore tested whether the effect size comparing asthma-cases to AMD-controls (OR_asthma_AMD_) was significantly different than the effect size when comparing the same asthma-cases to RA-controls (OR_asthma_RA_). Formally, assuming the effect sizes $\beta$=log(OR) are normally distributed, the difference between the effects sizes are also normally distributed:

$$N\left({\beta }_{asthma,AMD}-{\beta }_{asthma,RA }, {\sigma }_{asthma,AMD}^{2}+{\sigma }_{asthma,RA}^{2}-2*corr({{\beta }_{asthma,AMD},\beta }_{asthma,RA})*{\sigma }_{asthma,AMD}*{\sigma }_{asthma,RA}\right)$$

where ${\sigma }_{asthma,AMD}^{2}$ and ${\sigma }_{asthma,RA}^{2}$ are the variances for the respective effect sizes and $corr({{\beta }_{asthma,AMD}},{\beta}_{asthma,RA})$ was previously derived^66^ and is approximated as:

$$corr({{\beta }_{asthma,AMD}},{\beta}_{asthma,RA})\approx \frac{\left({n}_{kl0}\sqrt{\frac{{n}_{k1}{n}_{l1}}{{n}_{k0}{n}_{l0}}}+{n}_{kl1}\sqrt{\frac{{n}_{k0}{n}_{l0}}{{n}_{k1}{n}_{l1}}}\right)}{\sqrt{{n}_{k}{n}_{l}}}$$

where n_k0_, n_k1_, and n_k_ are the number of controls, the number of cases and the total number of samples in the asthma versus AMD association and n_l0_, n_l1_ and n_l_ correspond to similar numbers for the asthma versus RA association. We observed that a conservative threshold of P < 0.01 filtered association signals known to be originating from our non-asthma controls, we therefore set this as our threshold for filtering suggestive association signals originating from one of the controls (and not our asthma cases). We note that this test cannot distinguish between variants associated with increased asthma risk from variants associated with both AMD and RA. In addition, pleiotropic variants between asthma and either control will have reduced significance in the case of similar effect and increased significance in the case of opposite effects.

We employed co-localization analysis to provide further support of a shared causal variant between two association signals. Co-localization analysis was carried out in R with the *coloc* software^25^. Input statistics included p-values generated in this study (after removing variants with a significant differential effects p-value, see above) and eQTL p-values generated by GTEx^67^ (v8). We used MAFs from our asthma GWAS study as additional input into the *coloc* (version 3.1) software. We used a 1 megabase window around the index variant in the colocalization analysis.

SNP-based heritability of common variants and genetic correlation between traits was estimated using LD-score regression (version 1.0.1)^21^. Asthma risk statistics from this study were used as input after filtering variants failing the differential effects test (see above). We additionally used publicly available summary statistics for lung function^29^, blood eosinophil count^30^, and a recently reported moderate-to-severe asthma risk GWAS^8^. We used pre-computed LD-scores from the European subset of 1000 Genomes^68^. We further filtered our input variants to those available in HapMap3 as recommended. We assumed a prevalence of 0.084 for asthma (https://www.cdc.gov/nchs/products/databriefs/db94.htm). We further provide h^2^ estimates for a range of prevalences in the Supplementary Note S1.

Age of onset data were available for the EXCELS, TENOR II, EXTRA and Q4458G cohorts, for a total of 1456 subjects. In the UK Biobank cohort, age of onset data were available for 5362 subjects (see above). We dichotomized age of onset into childhood onset asthma (≤ 12 years of age) and adult onset asthma (≥ 25 years of age)^69^. This resulted in 665 childhood onset and 791 adult onset asthma subjects in our study, and 1261 childhood onset and 4101 adult onset asthma in the UK Biobank study.

We used four publicly available summary statistics to create polygenic scores (PS) for asthma^2^, allergic disease^22^, lung function^29^ and blood eosinophil count^30^. For each PS, we first subset out variants that were present in both our study and the public dataset. We restricted our analyses to variants with MAF > 1% as measured in the European ancestry subset of the 1000 Genomes cohort. We additionally filtered out any variants which could have strand ambiguity (A/T, C/G), and any variants in the HLA region. We next clumped common variants using PLINK1.9 to find independently associated variants that were associated with the scoring trait at P < 5 × 10^–8^. Independent variants were defined as having pairwise low LD (r^2^ < 0.05) and were at least 1 Mb apart. Finally, individuals were scored in PLINK based on their genetic risk for each trait using the log(odds-ratio) of each SNP. See Supplementary Note S1 for an evaluation of how these PS performed.

Association analyses between risk scores and phenotypes were carried out via logistic regression in R (version 3.4.3). We corrected for genetic sex and the first five principal components.

Candidate genes for the candidate gene rare variant analysis were identified from previous studies. Specifically, we obtained 139 genes from Ferreira et al. study with either eQTL or coding level evidence linking a gene to the sentinel variant (see Supplementary Tables S12 and S14 in the original publication)^22^. We obtained 17 candidate genes from the moderate-to-severe asthma GWAS study that had eQTL support linking the gene to the sentinel variant (see supplementary Table S8 in the original publication)^8^. Finally, we obtained 73 lung function related candidate genes implicated by coding, eQTL or pQTL data from a recent lung function GWAS (see Table 1 in the original publication)^29^.

Permutation gene-set enrichment analysis was used to test for significant enrichment of rare variant burden in the three candidate gene gene-sets from common variant allergic disease, moderate-to-severe asthma, and lung function loci. Input genes with a p-value < 0.01 in the differential effects test were excluded from this analysis. We compared the observed sum of the -log_10_(p-values) for all genes in the gene-set to the observed sum of 5000 random samples from all genes tested. To ensure no biases were introduced by the number of rare variants tested in each gene, random genes were sampled to match the distribution of rare variants in the genes from each gene-set. A permutation-based p-value was calculated as the fraction of sums derived from random samples that were as or more significant than the observed sum.

### Ethics approval

All research in this study was conducted in accordance with the Declaration of Helsinki. The data used in this study were generated from clinical trial participants who signed informed consent forms approved by the ethics committee or IRB responsible for the country or site where the trial’s participants donated samples for research. Informed consent included use of these data for genetics research. Before execution of the study, an internal Genentech team of informed consent form experts reviewed the forms from all the studies to ensure appropriate use of the samples. The list of these ethics committee and/or IRBs is available in the Supplementary Note S1.

## Supplementary Information

Supplementary Information 1.

Supplementary Information 2.

## Data Availability

The moderate-to-severe asthma risk summary statistics generated during this study are available from the corresponding author on reasonable request.
