# Supplementary material for: A whole genome sequencing study of moderate to severe asthma identifies a lung function locus associated with asthma risk
Source: Sci Rep. 2022 Apr 2;12:5574. doi: 10.1038/s41598-022-09447-8 (PMC8976834; doi:10.1038/s41598-022-09447-8)
Supplement: Supplementary file 1 — Supplementary Information 1. [file 41598_2022_9447_MOESM1_ESM.docx]

# Supplementary Note

## Disease control design

To minimize batch effects we utilized age-related macular degeneration (AMD) and rheumatoid arthritis (RA) cases as controls in our study. Though this study design can be problematic if there is extensive genetic pleiotropy between these three traits. To investigate how genetically correlated these traits are, we utilized independent data derived from external RA, asthma and AMD GWAS^1-3^. We estimated pairwise shared heritability between all three traits via LD-score regression. We found no significant genetic correlation between the traits (see table below).

| **Trait pair** | **Genetic correlation (p-value)** |
| --- | --- |
| AMD-RA | 0.0345 (0.3223) |
| RA-asthma | 0.1173 (0.0596) |
| AMD-asthma | 0.0833 (0.0739) |

In our study, we employed a differential effects test to flag whether the effect size of associated loci differed when comparing our asthma cases to RA controls alone or AMD controls alone. This method fails when loci are associated with both RA and AMD. We therefore used GPA^4^ method to investigate whether specific loci were associated with both AMD and RA. We found two independent loci passing an FDR 0.05 (see below for a list of all variants in these loci), one of which was also significantly associated with asthma in our study. This locus has also been associated with asthma in previous studies^5,6^.

| **CHR** | **BP** | **SNP** | **P_AMD_** | **P_RA_** | **FDR** |
| --- | --- | --- | --- | --- | --- |
| 12 | 111538972 | rs648997 | 2.83x10^-5^ | 4.50x10^-5^ | 0.02417721 |
| 12 | 111585263 | rs11065961 | 3.24x10^-5^ | 4.30 x10^-5^ | 0.02592704 |
| 12 | 111556082 | rs593226 | 3.92x10^-5^ | 3.60 x10^-5^ | 0.02792692 |
| 12 | 111511590 | rs678436 | 3.67x10^-5^ | 4.30x10^-5^ | 0.02812676 |
| 14 | 68293424 | rs1950897 | 4.33x10^-10^ | 2.50x10^-7^ | 0.00012059 |
| 14 | 68283210 | rs3784099 | 3.28x^-10^ | 2.70x10^-7^ | 0.00012818 |
| 14 | 68287978 | rs1885013 | 7.18x^-10^ | 2.70x10^-7^ | 0.00012857 |
| 14 | 68287700 | rs2104047 | 7.41x^-10^ | 2.70x10^-7^ | 0.00012859 |
| 14 | 68286876 | rs911263 | 7.55x^-10^ | 2.80x10^-7^ | 0.00013241 |
| 14 | 68285926 | rs8008961 | 2.11x^-9^ | 4.30x10^-7^ | 0.0001881 |
| 14 | 68286570 | rs2208397 | 2.19x^-9^ | 4.40x10^-7^ | 0.00019166 |
| 14 | 68281151 | rs7148416 | 5.10x^-10^ | 5.30x10^-7^ | 0.00022077 |
| 14 | 68276590 | rs10131490 | 9.04x^-10^ | 9.00x10^-7^ | 0.0003385 |
| 14 | 68272584 | rs8015139 | 5.54x^-10^ | 2.60x10^-6^ | 0.00079472 |
| 14 | 68274207 | rs7148882 | 5.47x^-10^ | 2.70x10^-6^ | 0.00081924 |
| 14 | 68291042 | rs7155473 | 2.88x^-10^ | 1.10x10^-5^ | 0.00253861 |
| 14 | 68261708 | rs28498223 | 1.35x^-9^ | 3.10x10^-5^ | 0.00583801 |
| 14 | 68262507 | rs1957570 | 1.40x^-9^ | 3.10x10^-5^ | 0.00583806 |
| 14 | 68261762 | rs17105278 | 2.28x^-9^ | 3.10x10^-5^ | 0.00583898 |
| 14 | 68294269 | rs2877455 | 2.91x^-10^ | 2.20x10^-4^ | 0.02774241 |
| 14 | 68295488 | rs11158728 | 1.77x-^-10^ | 2.40x10^-4^ | 0.02969986 |
| 14 | 68302799 | rs12890167 | 2.61x^-10^ | 3.00x10^-4^ | 0.03535161 |
| 14 | 68303314 | rs12885750 | 2.73x^-10^ | 3.00x10^-4^ | 0.03535163 |
| 14 | 68297537 | rs12880842 | 3.35x^-10^ | 3.60x10^-4^ | 0.04072584 |

For loci significantly associated with asthma risk in this study (see Table 1), we found that the risk allele frequency (RAF) in non-Finnish Europeans in gnomAD (v.2.1.1) is more aligned with the RAF in controls than in cases. The only exception was for the association on chromosome 14, which we show above is a pleiotropic locus associated with asthma, RA, and AMD.

| **SNP** | **CHR** | **BP (GRCh38)** | **Nearest gene** | **Risk/non-risk allele** | **RAF_case_** | **RAF_control_** | **RAF_gnomAD_** |
| --- | --- | --- | --- | --- | --- | --- | --- |
| rs139210940 | 2 | 102265885 | *IL1RL2* | AT/A | 0.879 | 0.847, 0.843 | 0.855 |
| rs10455025 | 5 | 111069301 | *TSLP* | C/A | 0.387 | 0.34, 0.326 | 0.319 |
| rs17205170 | 6 | 32634706 | *HLA-DQA1* | G/T | 0.84 | 0.797, 0.779 | 0.798* |
| rs2875584 | 6 | 90240909 | *BACH2* | C/T | 0.706 | 0.666, 0.653 | 0.682 |
| rs7130588 | 11 | 76559639 | *C11orf30* | G/A | 0.386 | 0.358, 0.332 | 0.343 |
| rs2104047 | 14 | 68287700 | *RAD51B* | T/C | 0.312 | 0.262, 0.274 | 0.279 |
| rs11631778 | 15 | 71314041 | *THSD4* | G/A | 0.354 | 0.305, 0.311 | 0.320 |
| rs7216558 | 17 | 39913818 | *GSDMB* | T/C | 0.545 | 0.479, 0.494 | 0.496 |

*RAF listed for rs17205170 was obtained from 1000 Genomes individuals of European ancestry as the RAF was unavailable in gnomAD (v2.1.1).

Given the low genetic correlation between these traits, similar allele frequencies between controls and an external reference population, and the small number of shared loci between RA and AMD, we believe the use of RA and AMD as controls in our study has a minimal impact on the results of this study.

## Sample batch effects

Though all samples in this study were all processed with a uniform bioinformatics pipeline, there were two main differences with regards to sequencing platform used to generate data for this study. First, while the majority of samples used in this study were sequenced through Human Longevity, a small subset of the asthma cases (individuals in the TENORII cohort) were sequenced at The Broad Institute. Second, two different chemistries were used to sequence the cohorts at Human Longevity (see Supplementary Table 12).

To assess whether this impacted the associations in the HLA region, we investigated whether the two variants in the HLA region associated with asthma remained (including 1 HLA signal that was filtered due to the differentials effect test) after removing the samples sequenced at the Broad and correcting for the chemistry batch for the remaining samples. Both variants remained genome-wide significant, including the variant (rs34434863) that was filtered due to the differentials effects test (see below).

| **CHR** | **SNP** | **BP** | **P** |
| --- | --- | --- | --- |
| 6 | rs34434863 | 32591896 | 8.34x10^-26^ |
| 6 | rs17205170 | 32634706 | 1.36x10^-13^ |

We performed a similar analysis for all variants outside the HLA region that were significantly associated with asthma in our study (Table 1). The majority of variants remained significantly associated with asthma (see Supplementary Table S12) with the largest p-value after batch correction equal to 2.25x10^-6^.

## Polygenic score (PS) evaluation

We evaluated the performance of each disease PS by applying them to an allergic disease^7^ GWAS and an asthma GWAS^2^ distinct from the GWAS we used to generate each PS. Using the summaryAUC^8^ method, we estimated the AUC for the allergic disease and asthma PS to be 0.582 (variance=3.44x10^-6^) and 0.595 (variance=1.86x10^-5^), respectively. We next estimated the spearman’s correlation between each quantitative PS (eosinophils, FEV_1_, FEV_1_/FVC, FVC, and PEF) and their measured value in the UK Biobank. All PS were significantly correlated with their measured values (P<1x10^-16^) with varying correlations (see table below). Together these results suggest the PS employed in this study are able to capture a phenotypic variability in these traits.

| **Trait** | **Spearman’s correlation** |
| --- | --- |
| Blood eosinophil cell count | 0.16 |
| FEV_1_ | 0.12 |
| FVC | 0.11 |
| PEF | 0.08 |
| FEV_1_/FVC | 0.23 |

# Supplementary Figures


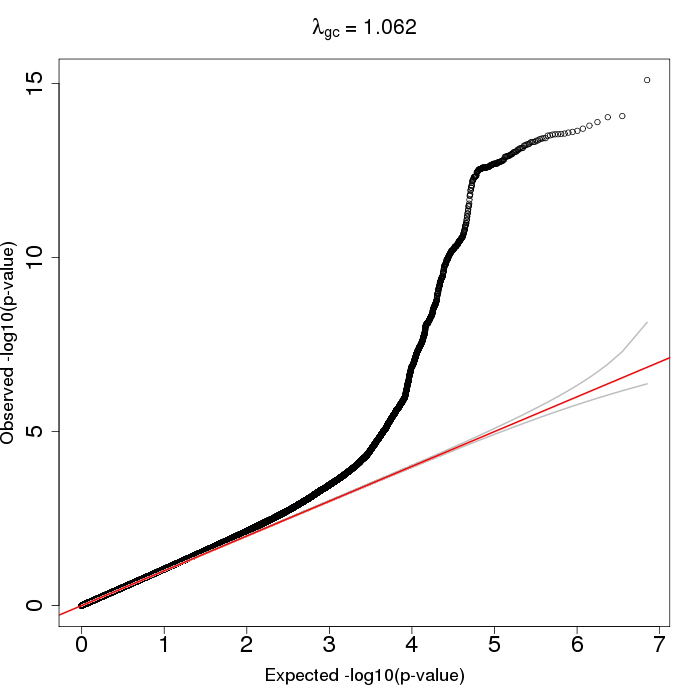


**Supplemental Figure 1.** QQ-plot of common variant associations (MAF>0.01) with variants failing the differential effects test removed.


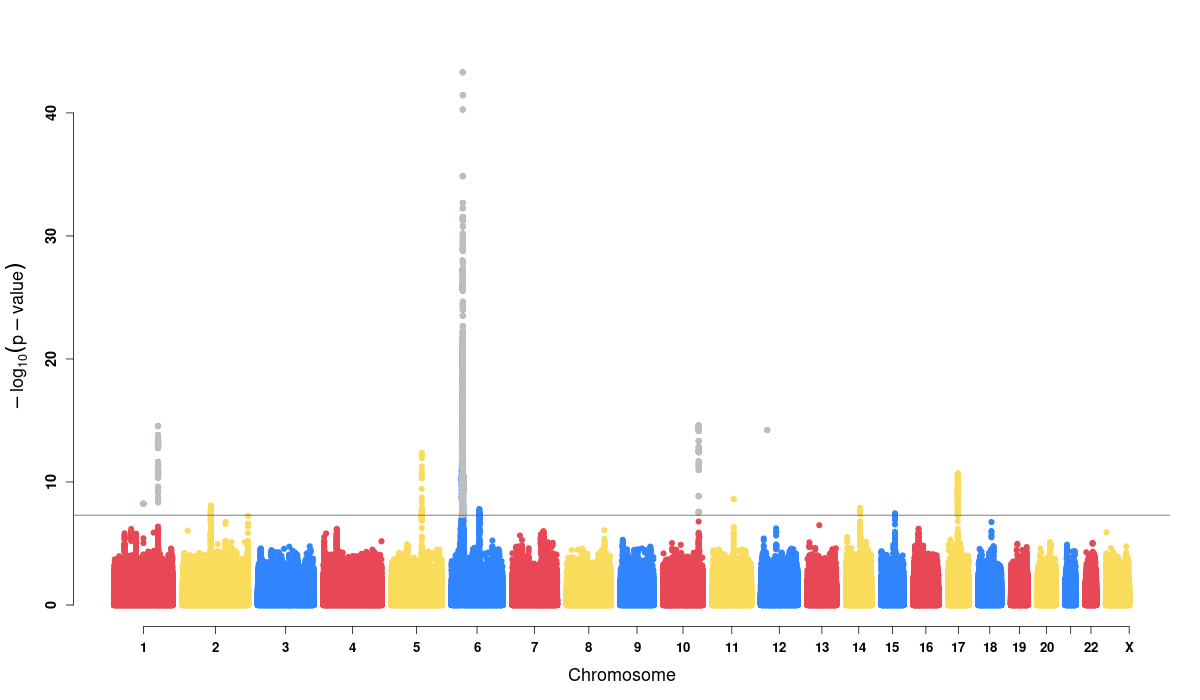


**Supplemental Figure 2.** Manhattan plot of all common variants (MAF>0.01) passing QC that were analyzed in this study. Variants reaching genome-wide significant (P<5x10^-8^), but did not pass the differential effects p-value threshold (P<0.01) are colored in grey.

**
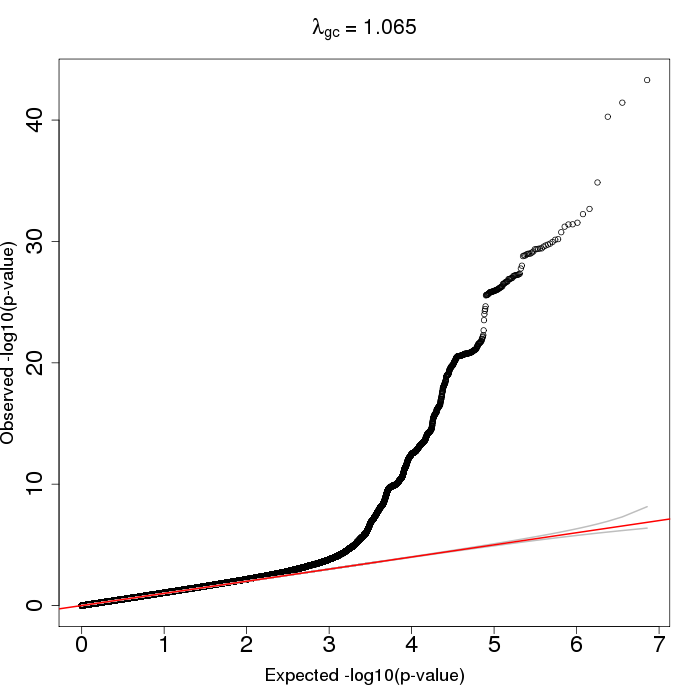
**

**Supplemental Figure 3.** QQ-plot of all common variants passing QC analyzed in this study.


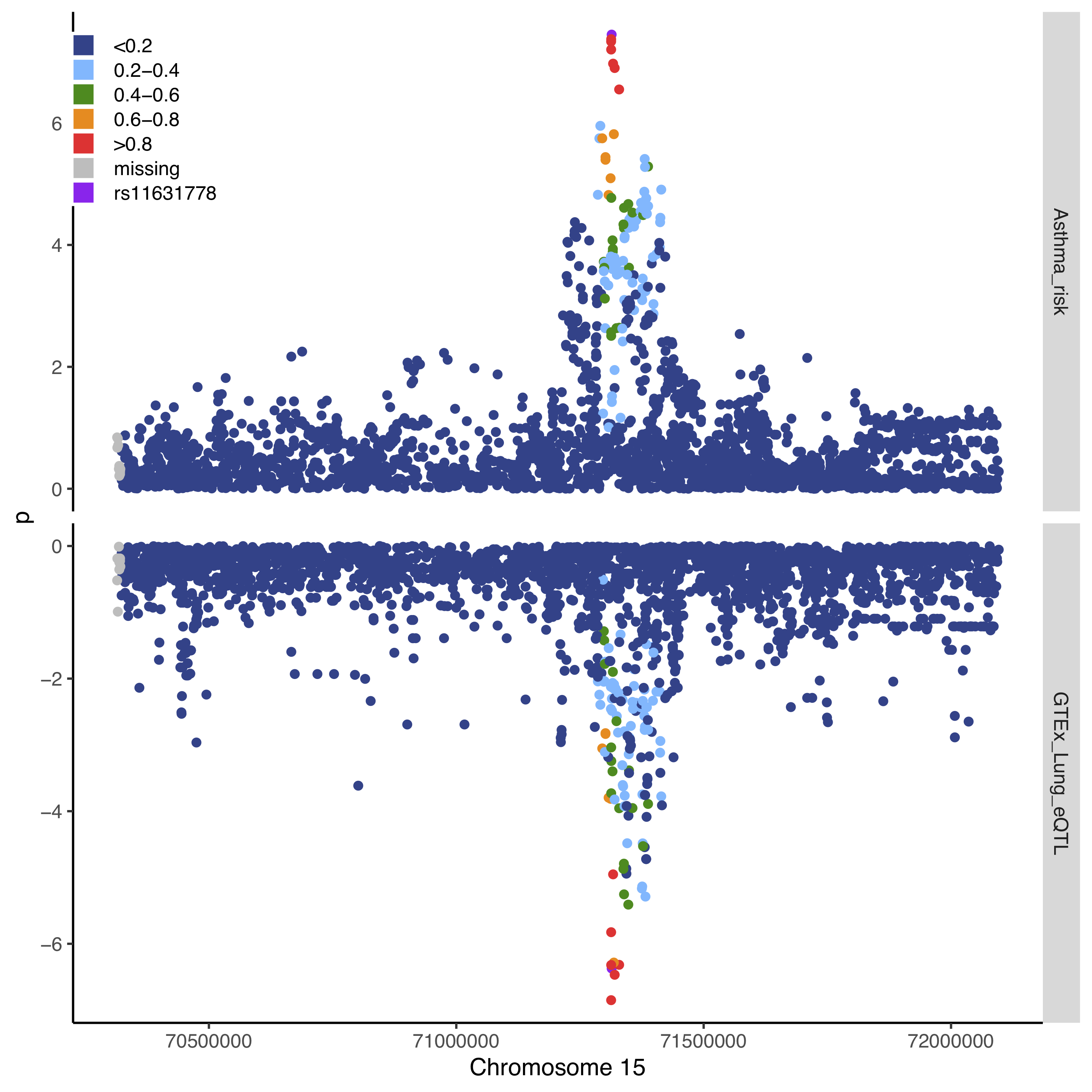


**Supplemental Figure 4.** Chicago plot for the associated region on chromosome 15 containing rs11631778. Top panel display the associations statistics for this study. The bottom panel displays association statistics for lung eQTL in GTEx v8.

**
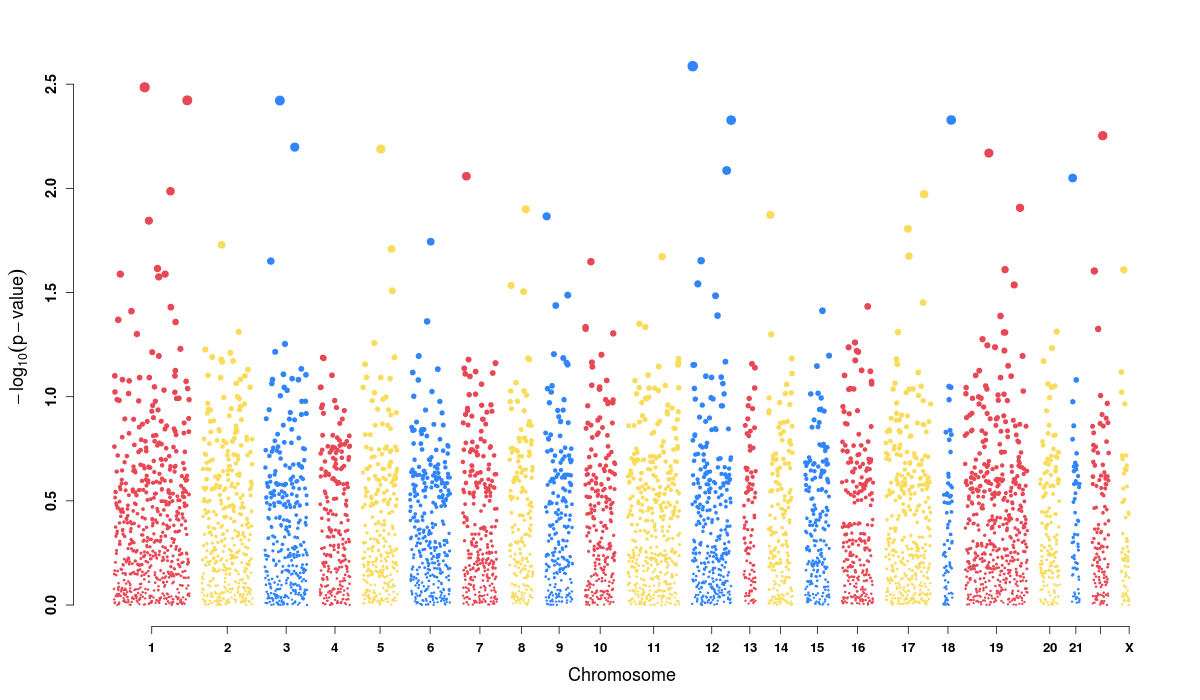
**

**Supplemental Figure 5**. Manhattan plot for gene burden test for rare predicted loss of function variants.

**
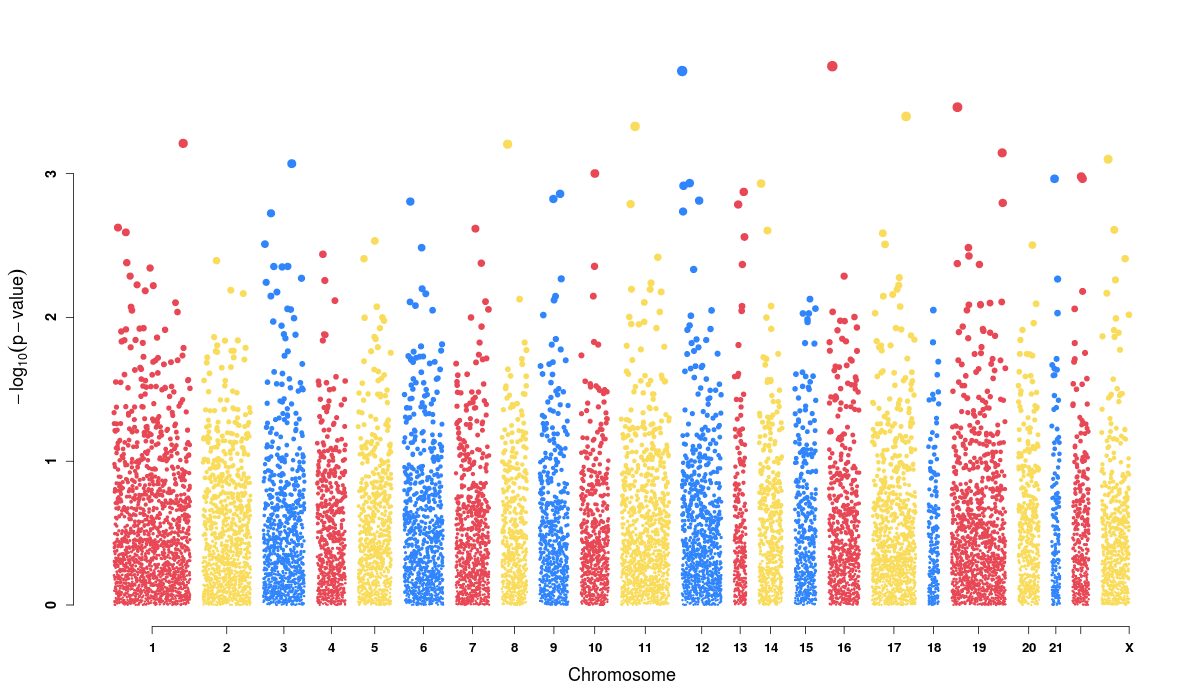
**

**Supplemental Figure 6.** Manhattan plot for gene burden test for rare predicted loss of function and moderate impact variants.

# List of IRBs and Ethics Committees

3RD Affiliated Hospital of Sys.Med.Sci.Uni | A Pesci Orvostudomanyi es Egeszsegtudomanyi Kozpont Regionalis Kutatas - Etikai Bizottsaga | Academic Medical Center Ethical Commission Academic Medical Center | Adelaide Health Services Ethics - (TQEH/LMH/MH | Administracion Nacional de Medicamentos Alimentos y Tecnologia Medica (ANMAT) | Administrative Panel on Human Subjects in Medical Research Stanford University Institutional Review Board | Agencia de EECC (CEIC) | Albert Einstein Healthcare Network Institutional Review Board | Asociacion Benefica Prisma | Assaf Harofe Medical Center | Auckland Ethics Committees Auckland Ethics Committees | Austrials Pty Ltd | Azienda Ospedaliera Universitaria | Barwon Health Research Ethics Committee | Base for drug clinical trail, Ethics committee of Anhui Medical University | Basic and Clinic Center in Studies Research Ethics I | Baylor College of Medicine and Affiliated Hospitals | Baylor College of Medicine IRB | Bellberry HREC | Bellberry Human Research Ethics Committee | Biomedical Institutional Review Board Chapel Hill | Biomedical Research Alliance of New York, L.L.C. Institutional Review Board | Biomedical Research Alliance of NY | Biomedical Research Ethics Board, University of Manitoba | Biomedical Research Ethics Committee University of KwaZulu-Natal | Brokenshire Ethics Committee / Institional Review Board | C. Enseñanza Invest Y Etica Hosp Privado San Jose | Cabrini human research Ethics Committee | Cabrini Human Research Ethics Committee, Cabrini Institute | CAEC-CEIC Autonomico de Ensayos Clinicos Andalucia Autonómico de ensayos clinicos de Andalucia | Cairns & Hinterland Health Service District Ethics Committee | Cambridge University Hospitals NHS Trust | Canterbury Respiratory Research Group | Cappesq | CCPPRB De Montpellier Saint Eloi | CCPPRB de Paris Saint Louis | CCPPRB Nimes UFR de Medecine | Ce ao oirm/s. Anna di torino | CE AO Universitaria S. Martino Di Genova | CE Aziendale dell'A.O. S. | CE CHU Sart Tilman | CE Dell'Azienda Oseda li Ruiniti Bergamo | Ce Locale Per La Sperim. Clin. Dei Medicinali Dell'azienda Osped.A Univer.A Senese Di Siena | CE ULB Erasme Bruxelles | Cebu Doctors' University - Cebu Doctors' University Hospital Institutional Ethics Review Committee | CEI FACOLTA MEDICINA E CHIRURGIA UNIV.STUDI UD | CEIC - Comissão de Ética para Investigação Clínica | CEIC de Andalucia (CCEIBA) | CEIC de Galicia (CAEI) | CEIC Hospital Clinic de Barcelona | CEIC Hospital La Paz | CEIC Hospital Puerta de Hierro Majadahonda | CEIC Hospital Unversitario Virgen Macarena | CEIC Intituto de Ciencias Medicas | Center Hospital of the National Center for Global Healthy and Medicine IRB | Central Health and Disability Ethics Committee | Centro De Estudios De Inv. Basica Y Clinica | CEQ of MI Dnipropetrovsk Clinical Association of Emergency Care of Dnipropetrovsk Regional Council | CEQ of Municipal Institution of Zaporizhzhia City of Multifield Clinical Hospital | CEQ of Si "National Institute of Phthisiology and Pulmonology" | CEQ of Si National Institute of Therapy | Chaim Sheba Medical Center | Charité - Universitaetsmedizin Berlin Ethikkommission | Chesapeake Research Review, Inc. | Chonnam National University Hospital IRB | CIADS Research Co Ltd | CIEC Fundacio Unio Catalana D'Hospitals | Cleveland Clinic Foundation IRB | Clinical Center of Serbia | Clinical Hospital Centre Zemun | Clinical Research Ethics Committee of Cork Teaching Hospitals | CMO St. Radboud Huispost | College of Physcians and Sugeons of Alberta Research Ethics Review Committee | Colorado Multiple Institutional Review Board | Comision Conjunta de Investigacion en Salud (CCIS) | Comision de Ética del Hospital General de Mexico | Comision de Investigacion y Etica Centro Medico Nova | Comissão de Ética para a Saúde | Comissão de Ética para análisa de projeto de pesquisa do Hospital das Clínicas da Faculdade de Medicina da USP - (CAPPESQ) | Comissao Nacional de Etica em Pesquisa - CONEP | Comission on Ethics Questions of MI of Health Care City Clinical Hospital | Comitato di Bioetica - Policlinico San Matteo | Comitato Di Bioetica Azienda Ospedaliera Papa Giovanni XXII | Comitato Di Bioetica Azienda Policlinico Universitario di Messina Via Consolare Valeria | Comitato di Bioetica Fondazione IRCCS Policlinico S. Matteo di Pavia | Comitato di Bioetica Policlinico S. Matteo | Comitato etico Az. Policlinico Umberto I Roma | Comitato Etico A.O. Istituto Ortopedico Gaetano Pini | Comitato Etico A.O. Luigi Sacco | Comitato Etico A.O. Ospedale Civile di Legnano | Comitato Etico A.U.O Policlinico P. Giacconec/o Direzione generale | Comitato Etico ASL 4 di L’Aquila | Comitato Etico Ausl3 Genovese | Comitato Etico Azienda Ospedaliera " Luigi Sacco" | Comitato Etico Azienda Ospedaliera "Spedali Civili di Brescia" | Comitato Etico Azienda Ospedaliera Arcispedale S. Anna | Comitato Etico Azienda Ospedaliera II Università di Napoli | Comitato Etico Azienda Ospedaliera Luigi Sacco | Comitato Etico Azienda Ospedaliera Ospedale S. Martino di Genova e Cliniche Universitarie Convenzionate | Comitato Etico Azienda Ospedaliera Ospedale San Carlo Di Potenza | Comitato Etico Azienda Ospedaliera Pisana-Toscana | Comitato Etico Azienda Ospedaliera Spedali Civili | Comitato Etico Azienda Ospedaliera Spedali Civili di Brescia | Comitato Etico Azienda Ospedaliero di Rilievo Nazional dei Colli di Napoli | Comitato Etico Azienda Policlinico Umberto I, | Comitato Etico Catania 1 presso A.O. Universitaria Policlinico Vittorio Emanuele | Comitato Etico Del Dimi (Dipartimento Di Medicina Interna E Specialita' Mediche) Dell'universita' Di Genova - Ospedale S.Martino Cambridge Local Research Ethics Committee | Comitato Etico dell' Azienda Ospedaliera Carreggi | Comitato Etico Dell'az. Osped.A Univ.A Policlinico P. Giaccone Dell'univ. Di Palermo | Comitato Etico Dell'az. Osped.A-Univ.A Della Ii Universita' Degli Studi Di Napoli | Comitato Etico dell'Azienda Ospedaliera "Arcispedale Sant'Anna" di Ferrara Comitato Etico Locale per la Sperimentazione Clinica dell'Ospedale L. Sacco di Milano | Comitato Etico Dell'azienda Ospedaliera Universitaria San Martino Di Genova | Comitato Etico dell'Azienda Ospedaliero Universita | Comitato Etico Dell'irccs Istituti Ortopedici Rizzoli | Comitato Etico dell’Azienda | Comitato Etico dell’azienda Ospedaliera Senese Centro Didattico Le Scotte | Comitato Etico Della Asl To/2 Di Torino | Comitato Etico Della Ausl 4 Di L'aquila | Comitato Etico Della Provincia di Ferrara | Comitato Etico Dipartimento Di Medicina Interna Universita' Degli Studi Di Genova | Comitato Etico Indipendente del Policlinico di Udine | Comitato Etico Indipendente Del Policlinico Universitario Di Udine | Comitato Etico Indipendente Di Etica Dell'azienda Ospedaliera "Carlo Poma" Di Mantova | Comitato Etico Interaziendale Delle Aso Oirm/S. Anna E Ordine Mauriziano Di Torino | Comitato Etico Istituto Di Farmacologia Policlinico Universitario Di Udine | Comitato Etico Locale per la sperimentazione clinica dell'Azienda Ospedaliera Luigi Sacco di Milano | Comitato Etico Milano Area C presso Azienda Ospedaliera Niguarda | Comitato Etico Ospedale di Circolo e Fondazione Macchi | Comitato Etico per la Sperimentazione | Comitato Etico per la sperimentazione Azienda Ospedaliera di Padova | Comitato Etico Per La Sperimentazione Dell'azienda Ospedaliera "Istituti Ospedalieri Di Verona" | Comitato Etico Per La Valutazione E Controllo Delle Sper.Clin. Dei Medicinali Dell'ausl 4 Di Prato | Comitato Etico Per Lo Studio Del Farmaco Sull'uomo | Comitato Etico per lo Studio del Farmaco sullUomo Azienda Ospedaliero- Univesitaria Pisana | Comitato Etico Policlinico IRCCS San Matteo | Comitato Etico Policlinico Universitario | Comitato Etico Provinciale di Modena Presso Azienda Pspedaliero Universitaria Policlinico di Modena | Comitato Etico Provinciale Di Reggio Emilia | Comitato Etico Universita' Cattolica S. Cuore Policlinico Gemelli | Comitato EticoUniversità degli Studi Federico II | Comitato Per La Sperim. Clin. Dei Medicinali Dell'azienda Ospedaliero- Universitaria Pisana Di Pisa | Comitato Per La Sperimentazione Clinica Dei Medicinali | Comité Autonomico de Ensayos Clínicos de Anadalucia(CAEC) | Comité Autonómico de Ensayos Clínicos de Andalucía(CAEC) | Comite Bioético de la Unidad de Investigacion en Enfermedades Cronico Degenerativas S.C | Comité Bioético Para La Investigación Clínica | Comite Bioético para la Investigación Clínica (CBIC) | Comité Bioético para la Investigación Clínica S.C. | Comite Consultatif de Protection des Personnas | Comite Consultatif de Protection des Personnas dans la Recherche Biomedicale | Comite Coordinador de Etica de la Investigacion Biomedica de Andalucia | Comité d'Ethique de la Faculté de Médecine | Comite d'Ethique de la recherche de l'Hopital de Maisonneuve Rosemont | Comité d'Ethique ULB Erasme | Comite de Bioetica de la Unidad de Investigacion en Enfermedades Cronico Degenerativas S. C | Comite de Bioetica del Instituto de Rehabilitacion Psicofisica | Comité de Bioética del Instituto Nacional de Cardiología | Comite de Bioetica Institucional Communitario Municipalidad de Vicente | Comite De Bioetica IREP | Comite de Bioetica Unidad de Investigacion en Enfermedades Cronico Degenerativas | Comité de Bioetica- Hospital General de Agudos "Carlos G Durand" | Comité de Bioética. Unidad de Investigación en Enfermedades Crónico Degenerativas SC | Comite de Bioteca de la Unidad de Investigacion en Enfermedades cronico | Comité de Docencia e Investigacíon - Centro Médico Privado de Reumatología | Comite de Docencia e Investigacion Centro Privado | Comité de Docencia e Investigación d | Comite de Docencia e Investigacion del Instituto de Rehabilitacion Psicofisica | Comite de Docencia e Investigacion Organizacion Medica de Investigacion | Comité de Docencia e Investigación- Orga nización Médica de Investigación | Comité de Enseñanza, Investigación y Ética | Comite de Etic CimByTA (Centro de Investigacion Medico Biologica y Terapia) | Comite de Etic en Investigacion de la Facultad de Medicina de la Uanl Y Hospital Universitario | Comité de Etica CAICI CIAE | Comite de Etica CAICI- CIAP | Comité de Etica CAYRE | Comite de Etica de CER Investigaciones Clinicas | Comite de Etica de la Investigacion | Comité de Etica de la Investigación Riesgo de Fractura S.A | Comite de Etica de PRISMA | Comité de Etica del Hospital CEM | Comité de Etica del Hospital Edgardo Rebagliati Martins-Essalud | Comité de Etica del Hospital Regional 1° de Octubre ISSSTE | Comité de Ética del Instituto Jalisciense de Investigacion Clínica S.A. de C.V. | Comite de Etica E Investigacion Christus Muguerza del Parque S.A. de C.V. | Comité de Ética e Investigación Comité Bioético para la Investigación Clínica | Comite de Etica e Investigacion del Hospital Universitario de Saltillo | Comite de Etica e Investigacion Hospital Aranda de la Parra | Comite de Etica e Investigacion Hospital Juarez de Mexico | Comité de Etica e Investigación Médica | Comite de Etica e Investigacion Unidad de Investigacion Clinica en Medicina | Comite de Etica em Pesquisa - HU CAS/UFJF | Comite de Etica em Pesquisa com Seres Humanos da Pontificia Universidade Catolica de Campinas | Comitê de Ética em Pesquisa da Faculdade de Ciências Médicas da UNICAMP | Comitê de Ética em Pesquisa da PUCRS | Comite de Etica em Pesquisa da PURCS | Comitê de Ética em Pesquisa da Universidade Federal de São Paulo / Hospital São Paulo | Comite de Etica em Pesquisa de UNIFESP | Comitê de Ética em Pesquisa do Complexo Hospitalar Heliópolis | Comite de Ética em pesquisa do Grupo Hospitalar Conceição CEP/GCP-GHC Ger6encia de Ensino e Pesquisa - Comitê de Ética em Pesquisa | Comitê de Ética em pesquisa do Grupo Hospitalar Conceição CEP/GCP/GHC | Comitê de Ética em Pesquisa do Hospital das Clinicas da Universidade Federal do Paraná - HCUFPR | Comite de Ética em Pesquisa do Hospital Geral de Goiania | Comitê de Ética em Pesquisa do Hospital Geral de Goiânia - CEPHGG | Comitê de Ética em Pesquisa do Hospital Israelita Albert Einstein | Comitê de Ética em Pesquisa do Hospital São Paulo/UNIFESP | Comite de Etica em Pesquisa do Hospital Universitario Pedro Ernesto | Comitê de Ética em Pesquisa em Seres Humanos da Irmandade da Santa Casa de Misericórdia da São Paulo. | Comite de Etica em Pesquisa em Seres Humanos do Hospital de Clinicas- HC/UFPR | Comitê de Ética em Pesquisa em Seres Humanos do Hospital Heliópolis | Comitê de Ética em Pesquisa em Seres Humanos do Hospital Universitário da Universidade Federal de Juiz de Fora | Comite de Etica em Pesquisa em Seres Humanos do Instituto de Assistencia Medica ao Servidor Publico Estadual / IAMSPE / SES | Comitê de Ética em Pesquisa em Seres Humanos do Instituto de Saúde e Bem Estar da Mulher - ISBEM/SP | Comitê de Ética em Pesquisa em Seres Humanos Hospital Universitário Pedro Ernesto- UERJ/RJ | Comitê de Ética em Pesquisa Hospital Heliópolis | Comitê de Ética em Pesquisa Hospital São Paulo | Comitê de Ética em Pesquisa Humana e Animal do Hospital Geral de Goiânia - CEPHA | Comite de Etica em Pesquisa Medica e Animal do Hospital das Clinicas da Universidade | Comitê de Ética em Pesquisa Prof. Celso Figueirôa | Comitê de Ética em Seres Humanos do Hospital das Clínicas/Universidade do Paraná | Comite de Etica en Farmacologia Clinica Fundacion CIDEA | Comité de Etica en Investigación (nuevo comité IREP) | Comite de Etica en Investigacion de Cemsi | Comité de Ética en investigación de CEMSI (Centro de Especialidades Médicas de Sinaloa) | Comité de Etica en Investigación del Hospital Guillermo Almenara Irigoyen Essalud | Comite de Etica en Investigacion Instituto de Investigaciones Clinicas | Comité de Ética Independiente Zugueme | Comite de Etica Pesquisa do Hospital Geral de Goiania | Comite de Etica y Investigacion del Hospital Angeles Lindavista | Comité de Etica-Comité de Docencia e Investigación, Instituto de Rehabilitación Psicofísica | Comité de Investigación Clinica Hospital Univeristaro La Paz | Comité de Investigación del Hospital Juárez de México | Comite de Investigacion para Estudios en Humanos | Comite de Investigacion y Etica del Hospital Christus Muguerza del Parque | Comite de Investigación y Etica del Hospital Juarez de Mexico | Comité de Investigación y Etica Hospital Universitario Comisión de Etica | Comité de Investigaciones y Etica en Investigaciones Hopital Pablo Tobón Uribe | Comité de Protection des Personnes Nord Ouest I | Comité Ético Autonómico de Ensayos Clínicos de Andalucía | Comite Etico Cientifico, Hospital CIMA San Jose Laboratory | Comite Etico de Investigacion Clinica | Comité Ético de Investigación Clínica - Cantabria | Comite Etico de Investigacion Clinica Ceic Galicia Edificio Administracion San Lazaro, s/n Spain | Comite Etico de Investigacion Clinica de Andalucia | Comite Etico de Investigacion Clinica de Galicia | Comité Ético de Investigación Clínica de Galicia | Comité Ético de Investigación Clínica del Hospital Regional Universitario Infanta Cristina | Comité Ético de Investigación Clínica del Hospital Universitario de Canarias | Comité Ético de Investigación Clínica del Hospital Universitario de La Princesa | Comité Ético de Investigación Clínica del Hospital Universitario de Valme | Comité Ético de Investigación Clínica del Hospital Universitario La Paz | Comite Etico de Investigacion Clinica Euskadi | Comité Ético de Investigación Clinica Hospital General Univeritario Gregorio Marañon | Comité Etico de Investigacion Clínica Hospital General Universitario de Guadalajara | Comite Etico de Investigacion Clinica Hospital General Universitario Gregorio Maranon | Comite Etico de Investigacion Clinica Hospital Regional Universitario "Infanta Cristina" | Comite Etico de Investigacion Clinica Hospital Universitario de Canarias | Comite Etico de Investigacion Clinica Hospital Universitario La Fe | Comite Etico dell'Aziend | Comite Indep. De Etica Para Ensayos En Farmacolog. | Comite Independiente de Etica e investigacion del Cnetro de Estudios de Investigacion Basica y Clinica S.C. | Comite Independiente de Etica para 202059 Ensayos en Farmacologia Clinica "Dr. Luis Maria Zieher" | Comite independiente de Etica para Ensayo en Farmacologia Clinica | Comite Independiente de Etica Para Ensayos en Farmacologia Clinica | Comite Institucional de Etica en Investigacion de la Asociacion Benefica Prisma | Comité Institucional de Etica en Investigación de la Asociación Benéfica Prisma | Comite Institucional de Etica en Investigacion de la Universidad San Martin | Comité Local de Investigacion Hospital de Especialidades centro Médico Nacional Comité de Investigación y Etica | Comite Nacional de Bioetica en Investigacion Instituto Conmemorativo Gorgas de Esudios de la Salud | Comitéde Ética - Instituto Reumatológico Strusberg | Commision on Ethics Qeustions of MI Cherkasy Regional Hospital of Cherkasy | Commission cantonale d'ethique de la recherche sur l'être humain | Commission centrale d'éthique, Hôpital Universitaire Genève | Commission d'Ethique de la Recherche clinique | Commission on Ethics Questions of 1-st City Clinical Hospital | Commission on Ethics Questions of Donetsk Municipal Treatment and Prophylactic Institution Central C | Commission on Ethics Questions of Municipal Institution Odesa Regional | Commission on Ethics Questions of Ukrainian Scientific and Research Institute of Invalid Rehabilita | Committee for Pharmaceutical Trials Faculty of Health Sciences Stellenbosch University | Committee for the Protection of Human Subjects Dartmouth College | Committee on Clinical Investigations Beth Israel Deaconess Medical Center | Committee on Clinical Investigations, Beth Israel Deaconess Medical Center | Committee on Human Rights Related to Research Involving Human Subjects | Committee on Human Rights Related to Researches Involving Subjects Faculty of Medicine, Ramathibodi Hospital, Mahidol University | Concord Repatriation General Hospital | CONEP - Comissão Nacional de Ética em Pesquisa, Esplanada dos Ministérios | CONEP - Comissao Nacional de Etica Pesquisa | Conjoint Health Research Board Faculty of Medicine University of Calgary | Conjoint Health Research Ethics Board | Conjoint Health Research Ethics Board Office of Medical Bioethics Heritage | Coordinador de Etica de la Investigacion Biomedica de Andalucia | Copernicus Group IRB | CPP de Brest | CPP IDF X (France) | CPP Sud Méditerrannée IV | CPP Sud Ouest et Outre Mer II | De Videnskabsetiske Komitéer for Region Hovedstaden | Debreceni Egyetem Orvos es Egeszsegtudomanyi Centrum | Department of Health, Pharmeceuticals registration and import/export control section | Domain Specific Review Board | Duke University Medical Center Institutional Review Board | EC - Arcispedale | EC Azienda Ospedaliera Luigi Sacco | EC Azienda Universitaria- Ospedaliera Senese | EC ern Seres Humanos do Hospital das Clinicas | EC of China Japan Friendship Hospital | EC of People's hospital of Peking University | EC of PUMC hospital | EC UZ Gent | Egeszsegugyi Tudomanyos Tanacs Tudomanyos es Kutatasetikai Bizottsag | EK Westfalen-Lippe ÄK | EK Würzburg | ETENE/Sub-Committee on Medical Research | Ethic Committee affiliated with State Institution of Healthcare "Republican Clinical Hospital n.a. V.A. Baranov" | Ethic Committee of NanJing GuLou hospital | Ethic Committee of ShangHai ChangHai hospital | Ethical & Independent Review Services | Ethical Clearance Committee on Human Rights Ramathibodi Hospital, | Ethical Clearance Committee on Human Rights Related to Researches Involving Human Subjects (Ramathibodi Hospital Mahidol University) | Ethical Review committee Royal Thai Army Medical | Ethical Review Committee Royal Thai Army Medical (Department Phramongkutklao Hospital and college of Medicine) | Ethics and Medical Research Committee St Vincent’s Hospital | Ethics Board of the Ministry of Healthcare and Social Development of the Russian Federation | Ethics Committee A.O. | Ethics Committee affiliated with Almazov Federal Heart, Blood and Endocrinology Centre of Department of Heath and Social Development of the Russian Federation | Ethics Committee affiliated with FSI "Republican Clincal Hospital n.a. G.G. Kuvatov" | Ethics Committee affiliated with Institution of Russian Medical Science Academy Scientific Research Institute of Rheumatology RAMS | Ethics Committee affiliated with SEI HPE "Kemerovo State Medical Academy of Federal Agency of Healthcare and social development" | Ethics Committee affiliated with SIH HPE "Ryazan Regional Clinical Cardiology Dispensary" | Ethics Committee Affiliated with SIH Ryazan Regional Clinical Cardiology Dispensary | Ethics Committee affiliated with St. Petersburg State institution of healthcare "Clinical rheumatologic hospital #25" | Ethics Committee affiliated with State Budget Educational Institution of Higher Education "St. Petersburg State Medical University n.a. acad. I.P. Pavlov of Department of Health and Social Development of Russian Federation" | Ethics Committee affiliated with State Budget Institution of Healthcare “Republican Clinical Hospital, n.a.G.G. Kuvatov” | Ethics Committee Affiliates with SIH “Clinical Rheumatologic hospital #25” | Ethics Committee at City Clinical Hospital | Ethics Committee at City Hospital | Ethics Committee at Clinical Hospital | Ethics Committee at Hospital of Saint | Ethics Committee at Moscow State Medical University | Ethics Committee at Municipal Polyclinic | Ethics Committee at Penza State University | Ethics Committee at Ryazan cardiologic | Ethics Committee at Saratove State Medical University | Ethics Committee at St. Petersburg State Medical University | Ethics Committee at the Institution of Russian Medical Science Academy Scientific Research Institute of Clinical and Experimental Lymphology of Siberian department RAMS | Ethics Committee at the Municipal institution of healthcare “Clinical Hospital #8 | Ethics Committee at the State Institution of Healthcare "Ulyanovsk Regional Clinical Hospital" | Ethics Committee at the State Institution of HealthCare of Yaroslav Region | Ethics Committee Barzilai Medical Center | Ethics Committee Faculty of Medicine Siriraj Hospital, Mahidol University | Ethics Committee Federal Body of Control of the Pharmaceutical Agents | Ethics Committee Fondazione Macchi | Ethics Committee For MultiCenter Trials (Bulgaria) | Ethics Committee for Multicenter Trials (Canada) | Ethics Committee of Bnei Zion Medical Center Haifa | Ethics Committee of General Hospital of PLA | Ethics Committee of Institute for the Development of Human Research Protections (IHRP) | Ethics committee of Kyiv City Tuberculosis | Ethics Committee of Qilu Hospital of Shandong University | Ethics Committee of Regensburg University Faculty of Medicine | Ethics Committee of State Burdenko Educational Institution of Higher Professional Education "Voronezh State Medical Academy n.a N.N. Burdenko of Ministry of Health care and Social development of Russia" | Ethics Committee of State Institution of Healthcare of Moscow "City clinical hospital No.20" | Ethics committee of The 3rd Affiliated Hospital of Sun Yat-sen University | Ethics Committee of the Faculty of Medicine, Institute of Forensic Medicine | Ethics Committee of the Khon Kaen University | Ethics Committee of the Medical Faculty of the University of Wurzburg | Ethics Committee on Internal Medicine of the Hospital District of Helsinki and Uusimaa | Ethics Committee Ospedale San Carlo di Potenza | Ethics Committee Revmatologický ústav | Ethics Committee Royal Perth Hospital | Ethics Committee, Faculty of Medicine Siriraj Hospital, Mahidol University | Ethics Committee, Hong Kong East Cluster | Ethics commmitee at City Clinical Hospital | Ethics Commmittee at Siberia State Medical University | Ethik-Kommission am Fachbereich Humanmedizin | Ethik-Kommission bei der Aerztekammer Niedersachsen | Ethik-Kommission bei der Landesaerztekammer Baden- Wuerttemberg | Ethik-Kommission bei der Saechsischen Landesaerztekammer | Ethik-Kommission der Aerztekammer Hamburg | Ethik-Kommission der Aerztekammer Nordrhein | Ethik-Kommission der Aerztekammer Westfalen- Lippe und der Medizinischen Fakultaet der Westfaelischen Wilhelms- | Ethik-Kommission der Arztekammer Nordrhein | Ethik-Kommission der Arztekammer Schleswig-Holstein (Ethik-Kommision I) | Ethik-Kommission der Medizinischen Fakultaet der Universitaet Wuerzburg | Ethik-Kommission der Medizinischen Fakultaet der Universitaet zu Kueln | Ethikkomission der Arztekammer | Ethikkomission der Landesarztekammer | Ethikkommission bei der Landesaerztekammer Baden-Wuerttemberg | Ethikkommission der Landesaerztekammer in Hessen | Ethikkommission der Medizinischen Fakultaet des Universitaetsklinikums Schleswig-Holstein (UKSH) Luebeck | Ethikkommission der Medizinischen Fakultaet Heidelberg | Ethikkommission der Medizinischen Universität Wien und des AKH der Stadt Wien | Ethikkommission der Stadt Wien | Ethikkommission des Kanton St. Gallen | Eticka komise Fakultni nemocnice Hradec | Eticka Komise IKEM a Thomayerovy | Eticka Komise Pro Multicentricka Hodnoceni | Eticka Komise Vseobecne fakultni nemocnice | Etická komisia | Eticka komisia Bratislavskeho | Etická Komisia Fakultnej | Eticka komisia Nitrianskeho | Eticka komisia pri Narodnom ustave reumatickych chorob | Etisch Comité Universitair Ziekenhuis Gent | Ewha Woman's University Mokdong | Faculté de biologie et de echerch Commission d’ethique de la eecherché clinique | Faculty of Health Sciences Research Ethics Committee, University of Pretoria | Fujita Health University Banbuntane Hotokukai Hospital Institutional Review | Geisinger IRB | Geisnger Health System Institutional Review Board | Gerencia de Ensino e Pesquisa - GEP Comitê de Ética em Pesquisa- CEP | Greenlane Clinical Centre | Grupo de Pesquisa e Pos Graduacao - Comissao Cientifica e Comissao de Pesquisa e Etica em Saude | Gunderson Clinic, Ltd. Human Subjects Committee | Gyogyszereszeti es Egeszsegugyi Minoseg- es Szervezetfejlesztesi Intezet | Hadassah University Hospital Local EC | Hamilton Health Sciences REB Henderson Hospital | Health Research Ethic Authority | Health Sciences Faculty, Research Ethics Committee University of Cape Town | Helsinki Committee Assaf-Harofe Medical Center | Helsinki Committee Barzilai Medical Center | Helsinki Committee Bnai Zion Medical Center | Helsinki Committee Chaim Sheba Medical Center | Helsinki Committee Hadassah Medical Center | Helsinki Committee Rabin Medical Center | Helsinki Committee Rambam Medical Center | Helsinki committee Soroka M.C | Helsinki committee Sourasky Medical Center | Helsinki Committee The Nazareth Hospital EMMS | Helsinki Committee-Haemek EC/IRB | Helsinky Committee Sourasky Medical Center | HKEC Ethics Committee | HKU/HA HKW IRB | Hong Kong / Hospital Authority Hong Kong West Cluster (HKU/HA HKW IRB) | Horoshima Allergy and Respiratory Clinic Institutinal Review Board | Hospital Angeles del Pedregal Comité de Bioetica | Hospital Clinico San Carlos | Hospital Clinico San Carlos Servicio de Farmacologia Clinica | Hospital Edgardo Rebagliati Martins-Essalud Eticki odbor Instituta za reumatologiju- Beograd | Hospital General de Mexico | HREC (Tasmania) Network | Human Research Ethics Committee (TQEH/LMH/MH) | Human Research Ethics Committee, Clinical Governance Unit, North Coast Area Health Service | Human Research Ethics Committee, Royal Brisbane Hospital | Human Research Ethics Committee, University of the Witwatersrand | Human Research Protection Program La Jolla Village Professional Center | Human Studies Committee Washington University School of Medicine | Human Subjects Protection Program | Hunter Area Research Ethics Commitee | HUS Sisätautien eettinen toimikunta Biomedicum Helsinki | HUS Tutkimuseettiset toimikunnat | Ibarakihigashi National Hospital IRB | Institute of Lung Diseases Bojbodina | Institution of Russian Medical Science Academy | Institutional Board of Research Associates Clinical Trial Development New York University School of Medicine | Institutional Board of Research Associates New York University School of Medicine, | Institutional Research and Ethics Committee Jose Reyes Mem Med Center, | Institutional Review Board Asian Hospital Medical Center | Institutional Review Board Chong Hua Hospital | Institutional Review Board for Baylor College of Medicine and Affiliated Hospitals | Institutional Review Board for Human Use University of Alabama | Institutional Review Board Medical Sciences Campus University of Puerto Rico | Institutional Review Board of Chang Gung Memorial Hospital | Institutional Review Board of Research Associates New York University School of Medicine | Institutional Review Board of the University of Hong Kong / Hospital Authority Hong Kong West Cluster (HKU/HA HKW IRB) | Institutional Review Board of the University of Hong Kong/Hospital Authority Hong Kong West Cluster (HKU/HA HKW IRB) | Institutional Review Board Presbyterian Hospital Of Dallas | Institutional Review Board Research and Biotechnology St. Luke's Medical Center | Institutional Review Board Research Review Committee/ Saint Vincent Hospital/ Fallon Community Health | Institutional Review Board Royal Thai Army Medicine Department | Institutional Review Board The Medical City | Institutional Review Board Tufts-New England Medical Center | Institutional Review Board, Oregon Health & Science University | Institutional Review Board/Research Committee/ Saint Vincent Hospital/Fallon Clinic | Instituto Conmemorativo Gorgas de Estudios de la Salud, Comité Nacional de Bioética de la Investigacíon | Interacademic Ethics Committee at the State budgetary educational Institution of higher professional education The First Moscow state medical university n.a. I.M. Sechenov under the Ministry of healthcare and social development of Russia Federation | IRB Services (Canada) | IRB Services (Institutional Review Board Services) (Canada) | IRB Services, Canada | Irmandade da Santa Casa de Misericórdia de São Paulo - Comitê de Ética em pesquisa em seres humanos | IUPUI and Clarian IRB | IUPUI and Clarian IRB Research & Sponsored | IUPUI and Clarian IRB Research & Sponsored Programs | IUPUI/CLARIAN Institutional Review Board | Iwata City Hospital Institutional Reivew | Joint Authority of the Hospital District of Helsinki and Uusimaa, Helsinki University Central Hospital, Ethics Committee, Department of Medicine | Joint CUHK-NTEC Clinical Research Ethics Committee | Joint The Chinese University of Hong Kong - New Territories East Cluster Clinical Research Ethics Committee | Joint Authority for the Hospital District of Helsinki and Uusimaa, HUCH department of Medicine, Ethics Committee | Juana Delgado Comité Ético de Investigación Clínica | Kantonale Ethikkommission Aargau | Kantonale Ethikkommission Bern | Kantonale Ethikkommission Zürich (KEK) | Karolinska Institute Regional Ethical Committee in Stockholm | Khonkaen University Ethics Committee for Human Research | Kindai University Hospital IRB | Kishiwada City Hospital IRB | Komisja Bioetyczna | Komisja Bioetyczna Okregowej Izby Lekarskiej w Lublinie | Komisja Bioetyczna przy Akademii Medycznej we Wroclawiu | Komisja Bioetyczna przy Instytucie Reumatologii | Komisja Bioetyczna przy Instytucie Reumatologii w Warszawie | Komisja Bioetyczna przy Okregowej Izbie Lekarskiej z siedziba w Bialymstoku | Komisja Bioetyczna przy Uniwersytecie Medycznym w Lodzi | Korean University Anam hospital IRB | Kumamoto Saishunsou National Hospital IRB | Kumamoto University Hospital IRB | Kyoto University Hospital IRB | Landesamt fuer Gesundheit und Soziales Berlin | Landesamt für Gesundheit und Soziales Berlin | Leeds (West) Research Ethics Committee | Lehigh Valley Hospital Institutional Review Board | Lithuanian Bioethics Committee | Local Ethics Committee at MHAT "St. Marina" | Local Ethics committee at St. Petersburg State Budge Instutition of Healthcare | Local Ethics Committee at UMHT "St. Anna" | Louisiana State University Health Sciences Center - Shreveport Institutional Review Board | Louisiana State University Health Sciences Center- Shreveport Institutional Review Board | LSU Health Sciences Center (LSU Medical Center) Institutional Review Board Sterling Institutional Review Board | Lung Intistute of Western Australia Inc | Machida Municpal Hospital IRB | Markusovszky Egyetemi Oktatokorhaz | Mayo Foundation Institutional Review Board | Medical Council of North Rhine | Medical Council of Saxony | Medical Ethics Committee | Medical Ethics Committee at Military Medical Academy | Medical Ethics Committee University of Malaya Medical Centre | Medical Reaearch Council Ethics Committee for Clinical Pharmacology | Medical Research and Ethics Committee Ministry of Health Malaysia | Medical Research and Ethics Committee Ministry of Health Malaysia, c/o Institute of Health Management | Medical Research Council, Ethics Committee for Clinical Pharmacology | Medisiininen eettinen toimikunta | Mercy Medical Center- Des Moines Instititional Review Committee | Mid Staffs Research & Development | Ministerio da Saude - Conselho Nacional de Saude - Comissao Nacional de Etica em Pesquisa - CONEP | Mount Sinai Hospital Research Ethics Board | Mount Sinai Hospital Research Ethics Board Western Institutional Review Board | Multi-Region Ethics Committee | Multi-region Ethics Committee (New Zealand) | Nagoya Ekisaikai Hospital IRB | NAP National Medical Ethics Committee | National Bioethics Committee for Medicine and Medical Devices | National Bioethics Committee Vegmuli | National Ethics Committee (Greece) | National Ethics Committee for Clinical Study in Medicine | National Ethics Committee Ministry of Health and Social Welfare | National Hospital Organization Fukuoka Nataional Hospital Institutional Review | New Territories West Cluster Clinical & Research Ethics Committee | Nihon University Hospital's Joint IRB | NMMC North Mississippi Health Services IRB | North Mississippi Health Services IRB | North Mississippi Medical center IRB | North Shore - Long Island Jewish Health System Institutional Review Board | North West MREC | North West Surrey LREC | Northern & Yorkshire MREC NHS Executive Northern & Yorkshire Department of Health John Snow House | Northern A health and Disability Ethics Committee | Northern University Office for the Protection of Research Subjects | Northwestern University Office for the Protection of Research Subjects Institutional Review Board | Nothwestern University Institutional Review Board | NRES Committee London - South East | NTT East Tohoku Hospital IRB | NTW Cluster Clinical & Research Ethics Committee | Office for Protection of Research Subjects | Office for the Protection of Research Subjects | Office of Protection of Research Subjects | Office of Research Ethics | Office of Research Ethics The University of Western Ontario | Office of Research Ethics University of Western Ontario | Office of Research Ethics Universtiy of Western Ontario Dental Sciences | Office of Research Ethics, University of Western Ontario | Okayama University Hospital IRB | Oklahoma Medical Research Foundation | OMI | Orszagos Reumatologiai es Fizioterapias Intezet Kutatasetikai Bizottsaga | Ospedaliera di Verona | P3 Research | Park Nicollet Health Services Park Nicollet Institute IRB | Park Nicollet Institutional Review Board | Parkway Independent Ethics Committee C/O Gleneagles Clinical Research Centre | Partners Human Research Committee | Pathology and Laboratory Medicine Mount Sinai Hospitial | Penn State College of Medicine IRB | Penticton Regional Hospital Ethics | Pharma Ethics (Pty) Ltd | Pharma-Ethics Independent Ethics Committee (South Africa) | Princess Alexandra Hospital | Protection of Rights of Human Subjects The University of North Carolina at Chapel Hill | Quorum Review IRB | R & D Department Royal Devon and Exeter Hospital | Rabin Medical Center Ethics Committee | Raigmore Hospital | Rambam Medical Center | Rambam Medical Center Ethics Committee | Redcliffe Caboolture Health Service District Human Resaerch Ethics committee | Redcliffe CabooltureHealthService EthicsCommittee | Redcliffe-Caboolture Health Service Ethics Committee | Regional komité for medisinsk forskningsetikk, Øst-Norge (REK Øst) | Regional komitè for medisinsk forskningsetikk, Region Øst (REK Øst) | Regional komitè for medisinsk og helsefaglig forskningsetikk | Regionala Etikprövningsnämnden | Regionala etikprovningsnamnden i Lund, avd 2 | Regionala Etikprövningsnämnden i Stockholm | Regionala Etikprovningsnamnden i Stockholm Karolinska Institutet | Research & Development Centre Northampton General Hospital NHS Trust | Research and Development Department East Sussez Hospital NHS Trust | Research and Development Department Mid Staffordshire NHS | Research and Development Department University Hospitals Coventry and Warwickshite NHS Trust University Hospital | Research and Development Department Wirral University Teaching Hospital NHS Foundation Trust | Research and Development Whipps Cross University Hospital | Research and Ethics Committee Repatriation General Hospital | Research Ethics Committee Chiang Mai University | Research Ethics Committee Faculty of Medicine | Research Ethics Committee, Faculty of Medicine, Chiang Mai University | Respiratory Clinical Trials Pty Ltd | Royal Brisbane and Women's Hospital Health Service District | Royal Brisbane Hospital and Royal Women's Hospital and Health Services District | Royal Hallamshire Hospital | Royal Perth Hospital Ethics Commitee | Royal Preseton Hospital | Rush University Medical Center Office of Research Affairs Institutional Review Board | Sagamihara Kyodo Hospital IRB | Saint Louis University IRB | Saint Vincent Hospital / Reliant Medical Group /Fallon Community Health Plan Institutional Review Board | SAVAHCS IRB | Schulman Associates | Scientific Board of the General Hospital of Athens "G. Genimatas" | Scientific Committee of the Euroclinic of Athens | Scientific Committee of the Sismanogleio General Hospital | Scientific Committee of University Hospital of Herakleion | Scientific Research Institute of Clinical Immunology of Siberian department RAMS | Sekino Hospital IRB | Selye Janos Korhaz es Rendelointezet | Sendai IRB | Seoul National University Hospital IRB | Service de Pharmacologie Clinique | Shropshire and Staffordshire LREC | Siriraj Institutional Review Board | Songklanagarind Ethics Committee | Soon Chun Hyang University Buchoen | Soroka Medical Center | South Birmingham LREC | South East Research Ethics Committee | South West Area Health Service HREC | Southern Adelaide Health Services / Flinders University Human Research Ethics Committee | Southern California Kaiser Permanente IRB | Southern Health Human Research Ethics Committee | Southern Health Human Research Ethics Committee A | Southern Health Human Research Ethics Committee Mount Hospital Ethics Committee | Southmead Hospital | SSWAHS Ethics Review Committee (RPAH Zone) | St Vincent's Campus - Human Research Ethics Committee | St. Alexis Medical Center IRB | St. John's Institutional Review Board | St. Joseph Mercy Health System Clincial Research Committee | St. Joseph’s Mercy Health Center IRB | St. Luke's Hospital IRB | Stanford University Institutional Review Board Administrative Panel on Human Subjects in Medical Research | Sterling Institutional Review Board | Sunnybrook and W omen’ s College Health Sciences Centre | Sunnybrook Health Sciences Centre | Sydney West Area Health Service (Westmead Campus) Human Research Ethics Committee | T.C Saglik Bakanligi Ilac Eczacilik Genel | Takamatsu Municipal Hospital IRB | Takatsuki Red Cross Hospital IRB | Tasmania Health and Medical Human Research Ethics Committee | Temple University Institutional Review Board | Thames Valley Multi-centre Research Ethics Committee | The Catholic University of Korea Soeul St Mary's Hospital IRB | The Chaim Sheba Medical Center EC | The Cleveland Clinic Foundation Institutional Review Board | The College of Physicians and Surgeo | The Ethic Committee of Beijing Union Medical College Hospital | The Ethic Committee of Beijing University Affiliated People's Hospital | The Ethic Committee of Shanghai Changhai Hospital | The Ethic Committee of Shanghai Renji Hospital | The Ethical Committee on Clinical Trials affiliated with State Budgetary Institution of Healthcare of Moscow City | The Ethical Review Committee for Research in Human Subjects Ministry of Public Health | The Ethics Committee (Rajavithi Hospital) | The Ethics Committee Faculty of Medicine Chulalongkorn University | The Ethics committee of Beijing China-Japan Friendship Hospital | The Ethics Committee of the Faculty of Medicine, Chulalongkorn University | The Ethics committee of the General Hospital of China PLA(301 Hospital) | The Ethics Committee of the Institution of Russian Medical Science Academy Scientific Research Institute of Rheumatology RAMS | The Ethics Committee Rajavithi Hospita | The Institutional Review Board of Taichung Veterans General Hospital | The Institutional Review Board of the Faculty of Medicine, Chulalongkorn University | The Milton S. Hershey Medical Center | The Sydney South West Area Health Service Ethics Review Committee | The University of Vermont Committee on Human Research | The University of Vermont Institutional Review Board | Tokyo-Eki Center-Buidling Clinic IRB | Toyama City Hospital Institutional Review | Trent Multi-centre Research Ethics Committee Laurie House | UHNS Research & Development | Unidad de Investigacion en Enfermedades Crónico degenerativas S.C. | Università Campus Bio Medico di Roma | Université de Lausanne Faculté de Médecine Commission de la Recherche Clinique | University Hospital of Brooklyn IRB | University Hospitals of Cleveland Institutional Review Board | University of Alabama at Birmingham Institutional Review Board for Human Use Committee For the Protection of Human Subjects | University of Arizona Human Subjects Protection Program | University of California San Diego Human Research Protection Program | University of Chicago Institutional Review Board | University of Florida Health and Science IRB | University of Medicine & Dentistry of New Jersey Robert Wood Johnson Medical School Robert Wood Johnson University Hospital Institutional Review Board | University of Pittsburgh Institutional Review Board | University of Saskatchewan Biomedical Research Ethics Board (Bio- REB) | University of Stellenbosch, Health Research Ethics Committee | University of Texas IRB | University of Utah Institutional Review Board | University of Utah Institutional Review Board Sterling Institutional Review Board | University of Washington Human Subjects Division | University of Wisconsin - Madison Health Sciences Human Subjects Committee | University of Wisconsin Health Sciences IRB | University of Witwatersrand Human Research Ethics Committee (medical) | University of Witwatersrand Human Research Ethics Committee Pharma-Ethics | Universtiy of Arizona Institutional Review Board | UNMHSC Human Research Review Committee | Uno Internal Medicine Respiratory Clinic IRB | Upper South A Ethics Committee | USC IRB | UZ Leuven - Commissie Medische Ethiek - toetsingscommissie | Vanderbilt University Institutional Review Board | Vanderbilt University IRB | Wake Forest University Health Sciences IRB | Wake Forest University IRB | Western Institutional Review Board | Winthrop IRB | WIRB | Yale University Human Research Protection Program | ZNA/OCMW Antwerpen Commissie voor Medische Etheik Secretariaat Neurologie

**REFERENCES**

1 Okada, Y. *et al.* Genetics of rheumatoid arthritis contributes to biology and drug discovery. *Nature* **506**, 376-381, doi:10.1038/nature12873 (2014).

2 Shrine, N. *et al.* Moderate-to-severe asthma in individuals of European ancestry: a genome-wide association study. *Lancet Respir Med* **7**, 20-34, doi:10.1016/S2213-2600(18)30389-8 (2019).

3 Fritsche, L. G. *et al.* A large genome-wide association study of age-related macular degeneration highlights contributions of rare and common variants. *Nature genetics* **48**, 134-143, doi:10.1038/ng.3448 (2016).

4 Chung, D., Yang, C., Li, C., Gelernter, J. & Zhao, H. GPA: a statistical approach to prioritizing GWAS results by integrating pleiotropy and annotation. *PLoS genetics* **10**, e1004787, doi:10.1371/journal.pgen.1004787 (2014).

5 Ferreira, M. A. R. *et al.* Genetic Architectures of Childhood- and Adult-Onset Asthma Are Partly Distinct. *American journal of human genetics* **104**, 665-684, doi:10.1016/j.ajhg.2019.02.022 (2019).

6 Pickrell, J. K. *et al.* Detection and interpretation of shared genetic influences on 42 human traits. *Nature genetics* **48**, 709-717, doi:10.1038/ng.3570 (2016).

7 Zhu, Z. *et al.* A genome-wide cross-trait analysis from UK Biobank highlights the shared genetic architecture of asthma and allergic diseases. *Nature genetics* **50**, 857-864, doi:10.1038/s41588-018-0121-0 (2018).

8 Song, L., Liu, A., Shi, J. & Molecular Genetics of Schizophrenia, C. SummaryAUC: a tool for evaluating the performance of polygenic risk prediction models in validation datasets with only summary level statistics. *Bioinformatics* **35**, 4038-4044, doi:10.1093/bioinformatics/btz176 (2019).
